# Unraveling Alveolar Fibroblast and Activated Myofibroblast Heterogeneity and Differentiation Trajectories During Lung Fibrosis Development and Resolution in Young and Old Mice

**DOI:** 10.1111/acel.14503

**Published:** 2025-02-13

**Authors:** Arun Lingampally, Marin Truchi, Xianrong Shi, Yuqing Zhou, Esmeralda Vasquez‐Pacheco, Georgios‐Dimitrios Panagiotidis, Stefan Hadzic, Janine Koepke, Ana Ivonne Vazquez‐Armendariz, Susanne Herold, Christos Samakovlis, Hector A. Cabrera‐Fuentes, Xuran Chu, Werner Seeger, Jin‐San Zhang, Elie El Agha, Bernard Mari, Saverio Bellusci, Chengshui Chen

**Affiliations:** ^1^ Department of Respiratory and Critical Care Medicine Quzhou People's Hospital, The Quzhou Affiliated Hospital of Wenzhou Medical University Quzhou Zhejiang China; ^2^ Department of Medicine II, Internal Medicine, Pulmonary and Critical Care Universities of Giessen and Marburg Lung Center (UGMLC), German Center for Lung Research (DZL), Justus‐Liebig University Giessen Giessen Germany; ^3^ Department of Medicine V, Internal Medicine, Infectious Diseases and Infection Control Universities of Giessen and Marburg Lung Center (UGMLC), German Center for Lung Research (DZL), Justus‐Liebig University Giessen Giessen Germany; ^4^ Cardio‐Pulmonary Institute (CPI) Giessen Germany; ^5^ Institute for Lung Health (ILH) Giessen Germany; ^6^ UMR CNRS 7275 Inserm 1323, IPMC, FHU‐OncoAge, IHU, RespiERA, Sophia Antipolis, Université Côte d'Azur Valbonne France; ^7^ Oujiang Laboratory (Zhejiang Lab for Regenerative Medicine, Vision and Brain Health), School of Pharmaceutical Science Wenzhou Medical University Wenzhou Zhejiang China; ^8^ Transdisciplinary Research Area Life and Health Organoid Biology, Life and Medical Sciences Institute, University of Bonn Bonn Germany; ^9^ Centro Interdisciplinario de Investigaciones Biológicas y Humanas (CIINBIOH) Universidad Autónoma Benito Juárez de Oaxaca Oaxaca Mexico; ^10^ Laboratory of Extracellular Lung Matrix Remodelling, Department of Internal Medicine Cardio‐Pulmonary Institute and Institute for Lung Health, Universities of Giessen and Marburg Lung Center (UGMLC), Member of the German Center for Lung Research (DZL), Justus‐Liebig University Giessen Giessen Germany

**Keywords:** activated myofibroblasts, aged mice, lineage‐tracing, lipofibroblast, pulmonary fibrosis, scRNAseq

## Abstract

Idiopathic pulmonary fibrosis (IPF) is an age‐associated disease characterized by the irreversible accumulation of excessive extracellular matrix components by activated myofibroblasts (aMYFs). Following bleomycin administration in young mice, fibrosis formation associated with efficient resolution takes place limiting the clinical relevance of this model for IPF. In this study, we used aged mice in combination with bleomycin administration to trigger enhanced fibrosis formation and delayed resolution as a more relevant model for IPF. Alveolosphere assays were carried out to compare the alveolar resident mesenchymal niche activity for AT2 stem cells in young versus old mice. Lineage tracing of the Acta2+ aMYFs in old mice exposed to bleomycin followed by scRNAseq of the lineage‐traced cells isolated during fibrosis formation and resolution was performed to delineate the heterogeneity of aMYFs during fibrosis formation and their fate during resolution. Integration of previously published similar scRNAseq results using young mice was carried out. Our results show that alveolar resident mesenchymal cells from old mice display decreased supporting activity for AT2 stem cells. Our findings suggest that the cellular and molecular mechanisms underlying the aMYFs formation and differentiation towards the Lipofibroblast phenotype are mostly conserved between young and old mice. In addition to persistent fibrotic signaling in aMYF from old mice during resolution, we also identified differences linked to interleukin signaling in old versus young alveolar fibroblast populations before and during bleomycin injury. Importantly, our work confirms the relevance of a subcluster of aMYFs in old mice that is potentially relevant for future management of IPF.

## Graphical abstract

Model of LIF‐to‐aMYF reversible switch during fibrosis formation and resolution in old mice. The main contributors during fibrosis formation to *Cthrc1*+ aMYF are Acta2+ LIF^high^ alveolar fibroblasts. During fibrosis resolution, *Cthrc1*+ aMYF differentiate back to the LIFhigh (please write "high" in superscript) alveolar fibroblasts cluster.

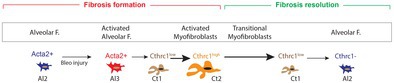

## 1 Introduction

Idiopathic pulmonary fibrosis (IPF) is characterized by the accumulation of excessive extracellular matrix (ECM) components due to an aberrant wound‐healing process. The initiation of IPF is widely accepted to start in the alveolar epithelium (Selman et al. 2001; Selman and Pardo 2006), either due to repetitive injury or aging. Chronic alveolar epithelium injury in the context of aging is proposed to lead to the activation of the adjacent mesenchyme, progressively resulting in the formation of the activated myofibroblasts (aMYFs).

The bleomycin (bleo) model is often used to study fibrosis in young mice. However, it is criticized as fibrosis formation is also associated with efficient resolution. In contrast, IPF in humans is a progressive disease which fibrosis never resolves. To overcome this caveat linked to the use of a single bleo administration in young mice, alternative approaches such as repetitive‐bleo injury in young mice have been proposed. Three successive weekly doses of bleo in young mice trigger increased fibrosis formation at 2 weeks. Subsequent fibrosis resolution, thought to be finalized at 4 weeks after a single bleo dose, is instead finalized at 6 weeks after the final dose (Chung et al. 2003). In addition, another study using eight biweekly bleo doses showed persistent fibrosis up to 10 weeks after the final dose (Degryse et al. 2010).

Another alternative approach to reflect human IPF is to use aged mice, which were reported to develop severe fibrosis with impaired resolution (Caporarello et al. 2020; Redente et al. 2011). Aging is associated with telomere shortening, cellular senescence, oxidative stress, and stem cell exhaustion (Bueno et al. 2020; Chanda et al. 2021; Cronkhite et al. 2008; Sgalla et al. 2018; Yanai et al. 2015). A recent study showed that the niche activity of the resident mesenchymal cells (rMCs aka alveolar fibroblasts) for the AT2 stem cells is lost within the aged mesenchyme due to increased expression of NADPH oxidase 4 (Nox4), an enzyme essential for regulating reactive oxygen species. The rMC‐AT2 stem cell niche activity is partially rescued with the partial loss of Nox4 in aged rMCs (Chanda et al. 2021). In addition, NOX4 is elevated in IPF lungs. Interestingly, siRNA‐based inhibition of *NOX4* led to a decrease in SMAD2/3 phosphorylation, which is downstream of Transforming growth factor beta (TGFβ) signaling, a master signaling cascade in IPF (Amara et al. 2010).

As aforementioned, the initiation of the IPF is proposed to start in the alveolar epithelium. Focusing on the epithelium, a recent study showed the effects of aging and injury on the alveolar epithelial type 2 (AT2) cells. This study observed that AT2s are significantly decreased in aged mice and that new AT2 subsets, enriched in the expression of inflammation, senescence, and apoptosis markers, emerge. Additionally, young AT2s increased the aging‐related markers and shifted towards these new AT2 subsets after bleo injury. However, young AT2s recovered back to their native state during fibrosis resolution. In contrast, aged AT2s failed to recover back during fibrosis resolution. This suggests that aging impairs the AT2 progenitors' renewal capability (Liang et al. 2023).

Considering the AT2s' role as progenitors, the Cell division control protein 42 homolog (Cdc‐42) is essential for the differentiation of AT2 cells to alveolar epithelial type 1 (AT1) cells. In mice, the deletion of *Cdc‐42* in AT2 cells at a young age triggered fibrosis formation from the periphery to the center of the lung with aging (Wu et al. 2020). These studies show that aged mice are primed for fibrosis compared to young mice. Recently, studies comparing young and old mice revealed that pathways associated with fibrosis are similarly regulated between young and aged mice. Aged mice, however, showed an increase in immune cells. Additionally, aged mice showed delayed fibrosis resolution, possibly due to decreased regenerative capacity of the aged lung (Klee et al. 2023; Weckerle et al. 2022).

Our previous studies showed that lipofibroblasts (LIFs), defined as resident mesenchymal cells located in the alveoli in proximity to alveolar type 2 epithelial cells (O'Hare and Sheridan 1970; Vaccaro and Brody 1978) are essential for the proliferation of AT2 stem cells (Taghizadeh et al. 2022; Taghizadeh et al. 2021). LIFs are also a potential source of aMYFs. Lineage tracing of LIFs and *Acta2* + aMYFs in the context of fibrosis formation and resolution in young mice demonstrated a LIF‐to‐aMYF reversible switch (El Agha et al. 2017). Recently, using lineage tracing of the *Acta2* + aMYFs associated with scRNASeq, we reported the cellular and molecular cues related to the LIF‐to‐aMYF reversible switch during fibrosis formation and resolution in young mice using the bleo model (Lingampally et al. 2024). We found that the aMYFs are heterogeneous and express Collagen triple helix repeat containing 1 (*Cthrc1*). Our data suggest that LIFs differentiate to *Cthrc1*+ aMYFs during fibrosis formation and switch back to their native LIF phenotype during resolution.

In contrast, whether a LIF‐to‐aMYF reversible switch takes place in aged mice is still unclear. To explore the LIF‐to‐aMYF reversible switch during aging, we employed 52–56‐week‐old *Tg(Acta2‐CreERT2)/+*; *tdTomato* ^*flox*^ mice, which replicate the mid‐age of humans where initiation of the fibrosis formation is proposed to take place (Jenkins et al. 2017).

This study used alveolosphere assays to compare the alveolar resident mesenchymal niche activity for AT2 stem cells in young versus old mice. Lineage tracing of the *Acta2* + aMYFs in old mice exposed to bleo followed by scRNA‐seq of the lineage‐traced cells isolated during fibrosis formation and resolution was performed to delineate the heterogeneity of aMYFs during fibrosis formation and their fate during resolution.

Our data indicate that the cellular and molecular bases of aMYFs formation and differentiation towards the LIF phenotype are conserved between young and old mice. Importantly, our work identifies a subcluster of aMYFs and alveolar fibroblasts that are potentially relevant for future management of IPF.

## 2 Material and Methods

### 2.1 Ethical Aspects and Mice

All animal experiments were performed according to the approved protocols by the Regierungspraesidium Giessen, the animal ethics committee of the University of Giessen (permit numbers: G57/2019–No.974_GP and G26/2020–No.1002_GP). All animals were housed under specific pathogen‐free (SPF) conditions with unlimited access to food and water. The room environment was maintained with 12 h of dark/light cycle at 22°C and 40%–70% humidity. *Tg(Acta2‐CreERT2)/+* mice (STOCK_*Tg(Acta2‐cre/ERT2)12Pcn*) (kind gift from Dr. Pierre Chambon, University of Strasbourg, France) were bred with *td‐Tomato* ^*flox*^ *(B6*; *129S6‐Gt (ROSA) 26Sortm9(CAG‐tdTomato)Hze/J)* (Jackson lab, 007909) to generate tamoxifen‐mediated reporter mice.

### 2.2 Bleo Instillation

52–56‐week‐old female mice were intratracheally (IT) subjected to either saline or bleo 2 U.kg^−1^ body weight (2 mg.kg^−1^) (Bleomedac, PNZ‐02411351). Lungs were harvested on days 14, 30, and 60 post intratracheal instillation for analysis.

### 2.3 Tamoxifen Administration

Tamoxifen (Sigma, T5648‐5G) was reconstituted in corn oil. Mice were intraperitoneally (IP) subjected (0.1 mg.g^−1^ body weight) with four injections on days 5, 7, 9, and 11 *after* saline or bleo instillation, respectively.

### 2.4 Immunofluorescence Staining

Mice were sacrificed, and the lungs were transcardially perfused with 10 mL PBS. Perfused lungs were fixed in 4% PFA and sequentially dehydrated with ethanol. The fixed‐dehydrated lungs were then embedded using paraffin. Five‐μm‐thick paraffin slices were deparaffinized and rehydrated, sequentially with xylol, 100%, 70%, 50%, and 30% ethanol, and finally in Milli‐Q water. The slices were then subjected to antigen retrieval by transferring the slices in citrate buffer and boiled for 15 min in a cooker. Following antigen retrieval, slices were cooled on ice for 20 min and washed with PBST three times. The slices were blocked with 3% BSA + 0.2% Triton‐X in PBS for 1 h. Slices were then incubated with primary antibodies: anti‐ α‐SMA/Acta2 (Sigma, F3777), anti‐tdTomato (Sicgen, AB8181‐200) at 4°C overnight. Following washing, slices were incubated with secondary antibodies for 1 h at room temperature and finally mounted with ProLong Gold Antifade Reagent containing DAPI (Molecular Probes, P36935).

### 2.5 RNAscope Assay

Five‐μm‐thick paraffin slices were used for the RNAscope assay. The assay was carried out according to the manufacturer's instructions (ACD, Doc. No. 323100‐USM) using the *Cthrc1*: 413341, *Inmt*: 486371‐C2, *Timp1*: 316841‐C3 and *Serpine2*: 435241‐C2 probes. Following the RNAscope assay, slides were blocked for 1 h with 3% BSA in PBS. Subsequently, the slides were then incubated overnight with anti‐Pro‐Col1a1 (Phosphosolutions, 321‐COLP) and anti‐tdTomato (Sicgen, AB8181‐200) at 4°C. After washing, the slides were incubated with the secondary antibody for 1 h at room temperature. Finally, the slides were counterstained with DAPI and mounted.

### 2.6 TUNEL Assay

Five‐μm‐thick paraffin slices were used for the TUNEL assay. The assay was carried out according to the manufacturer's instructions (Promega, G3250). Following the TUNEL assay, slices were blocked for 1 h with 3% BSA in PBS. Subsequently, the slides were then incubated overnight with anti‐tdTomato (Sicgen, AB8181‐200) at 4°C. After washing, the slides were incubated with the secondary antibody for 1 h at room temperature. Finally, the slides were counterstained with DAPI and mounted.

### 2.7 Hematoxylin and Eosin Staining

Five‐μm‐thick paraffin slices were deparaffinized and rehydrated. The sections were stained with hematoxylin (Roth, T865.2) for 2 min and eosin (Thermo Fisher Scientific, 6766007) for 1 min. Following staining, the slices were dried and mounted.

### 2.8 FACS Preparation

Mice were sacrificed and the lungs were transcardially perfused with 10 mL PBS. Lungs were finely chopped and digested in 0.5% collagenase type IV (Gibco 17104‐019) at 37°C for 45 min. Following incubation, the cell suspension is passed through 70 and 40 μm cell strainers. The single‐cell suspension was centrifuged, and the cell pellet was stained with anti‐Cd31 (Alexa Fluor 488‐conjugated 1:100, Biolegend, 102514), anti‐Cd45 (Alexa Fluor 488‐conjugated 1:100, Biolegend, 103122), anti‐Epcam (APC‐Cy7‐conjugated 1:50, Biolegend, 118217) and anti‐Sca1 (pacific blue‐conjugated 1:50, Biolegend, 108120) antibodies on ice for 20 min. After washing, SYTOX‐Blue (Invitrogen, S34857) was added to the cell suspension to exclude dead cells, and sorting was carried out using the FACSAria III cell sorter (BD Biosciences). Data were analyzed using FlowJo software (FlowJo LLC).

### 2.9 Generation of the scRNA‐Seq Data

Live sorted tdTomato+ cells were centrifuged and resuspended in 0.04% ultrapure BSA (Invitrogen, 01266574) in PBS for optimal cell concentration. The resuspended tdTom+ cells were loaded into the Chromium Controller (10× Genomics), and the cDNA libraries were prepared according to the manufacturer's instructions. Sequencing was performed by Nextseq2000 (Illumina Inc.), and reads were aligned against a custom mouse reference genome (mm10) and counted by STARsolo.

### 2.10 Analysis of the scRNA‐Seq Data

All downstream analyses were done with the Seurat R package (v4.1.0) (Hao et al. 2021). To be consistent with the cells annotation produced for the young Acta2+ mice dataset (Lingampally et al. 2024), the four old mice samples (saline d14, Bleo‐Tam d14, Bleo‐Tam d30, and Bleo‐Tam d60) were integrated with three young mice samples (saline d14, Bleo‐Tam d14, and Bleo‐Tam d60). First, the counts matrix of each sequenced sample were loaded as Seurat objects, then filtered using arbitrary thresholds for UMI, genes, and mitochondrial content (nCount_RNA < 30,000, nFeature_RNA > 800 and percent.mito < 0.15) before being concatenated. The concatenated matrix was normalized using the NormalizeData function. After normalization, the counts of the 2000 most variable features selected with “vst” method were scaled and centered before running PCA. Harmony (Korsunsky et al. 2019) was run on the PCA cell embedding, specifying the sample of origin as a covariate. kNN clustering was run on the first 50 dimensions computed with Harmony (using k.param = 10), same for UMAP visualizations in two‐dimensions. Based on UMI content and differentially expressed genes in each cluster, clusters corresponding either to remaining low‐quality cells or non‐mesenchymal cells were removed. The integration process was rerun on the subset of selected cells with the same parameters. Finally, cell clusters were annotated according to the expression of their specific markers described in (Tsukui et al. 2020). Subpopulations of *Cthrc1+* aMYFs, alveolar (LIF), adventitial, and peribronchial fibroblasts were identified by performing clustering on each particular cell subset, using the exact first 50 dimensions computed with Harmony on the complete dataset. Differential expressions between samples for a particular subpopulation were run using the FindMarkers function on normalized data.

### 2.11 Data Deposition

Single‐cell data have been deposited on the gene expression omnibus (GEO) repository: GSE253453, GSE221402, and GSE223664.

### 2.12 Alveolosphere Assay

Alveolosphere assay was carried out as described (Lingampally et al. 2024; Taghizadeh et al. 2021). The following mice were used to sort resident mesenchymal cells (rMCs) and Alveolar type 2 cells (AT2s): (1) rMCs from C57BL/6 mice were isolated from 8‐week‐old (young) and 35‐week‐old (mature) mice. rMCs from *Tg(Acta2‐CreERT2)/+*; *tdTomato* ^*flox*^ mice were isolated from 52 to 56‐week‐old (aged) mice. (2) tdTom^high^‐AT2s (These cells are *bona fide* AT2 cells and exhibit a significant capability for alveolosphere formation during homeostasis (Ahmadvand et al. 2021)) from *Sftpc* ^*CreERT2/+*^; *tdTomato* ^*flox*^ mice were isolated from 35‐week‐old mice. AT2s from C57BL/6 mice were isolated from 8‐week‐old mice.

In brief, sorted 50,000 rMCs and 5000 AT2s were reconstituted in 100 μL media and mixed in 100 μL ice cold Matrigel (Corning, 356231). The mixture was transferred to 24‐well 0.4 μm transwell inserts (Greiner bio‐one, 662641) and incubated at 37°C for 15 min for Matrigel polymerization. Next, 500 μL of media (sorting media plus 1% ITS (Gibco, 41400‐045)) was added to each well and incubated at 37°C in 5% CO_2_ for 14 days.

### 2.13 Statistical Analysis

For the comparison of two groups, unpaired, two‐tailed *t*‐tests were used. For comparisons involving more than two groups, one‐way ANOVA with *post hoc* Newman–Keuls multiple comparisons test or two‐way ANOVA with *post hoc* Šídák's multiple‐comparisons test was used. Values of *p* < 0.05 were considered statistically significant. All data are presented as mean ± SEM.

## 3 Results

### 3.1 Functional Characterization of the rMC Niche Activity for AT2 Stem Cells in Young and Aged Mice

The alveolosphere assay was carried out to determine the effect of aging on the rMC niche activity for AT2 stem cells. To generate organoids, rMCs Sca1+ from young (8‐week‐old) and mature (35‐week‐old) mice were isolated by FACS. rMCs Sca1+ are enriched in LIFs displaying the niche activity for AT2 stem cells (McQualter et al. 2013; Taghizadeh et al. 2021, 2022).

**FIGURE 1:**
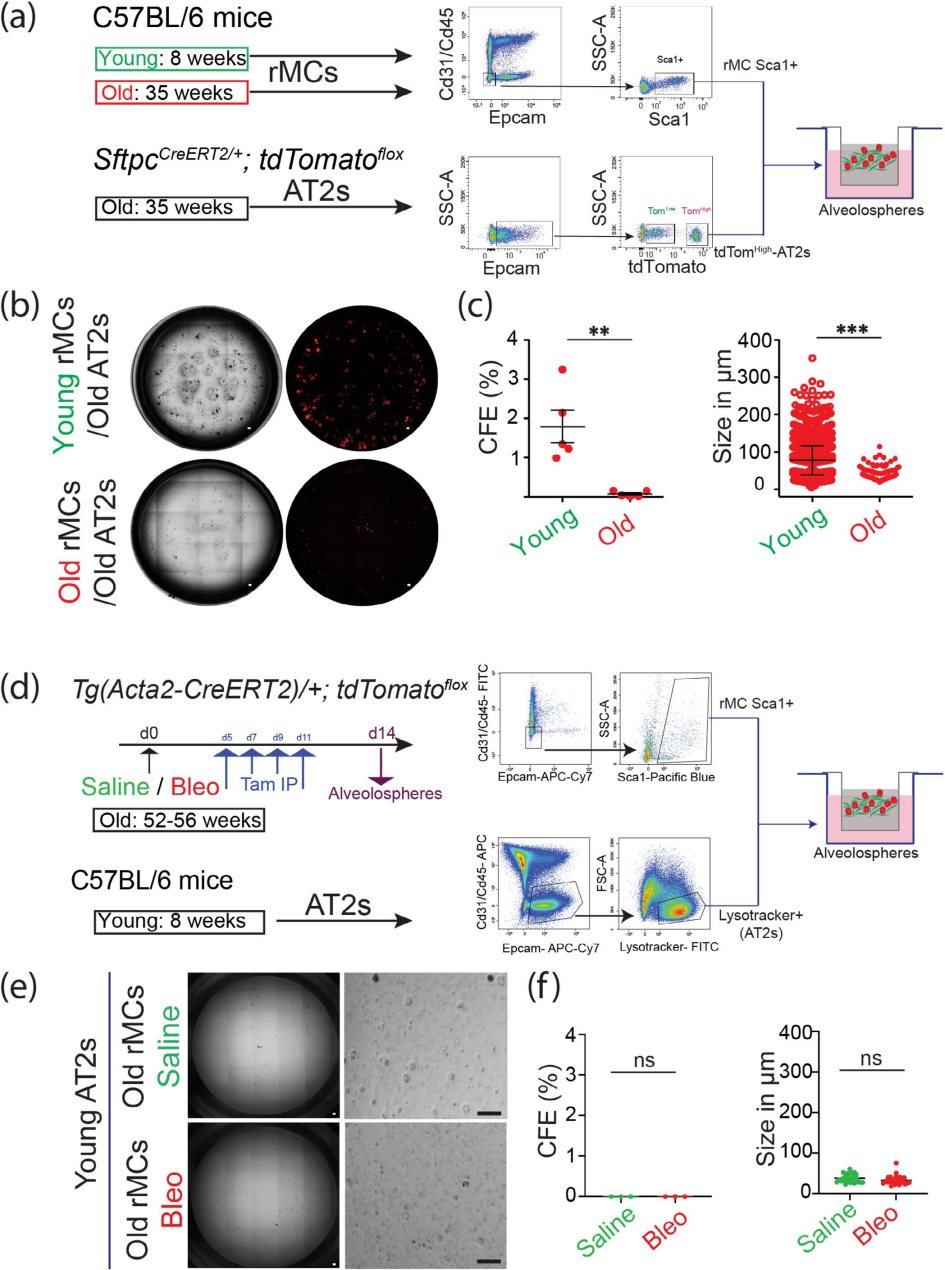
Evaluation of resident mesenchymal niche activity for AT2 stem cells upon aging. (a) Experimental design showing the rMCs (resident mesenchymal cells) isolated either from 8‐ or 35‐week C57BL/6 mice and gating strategy to sort Cd31/Cd45/Epcam triple‐negative (rMC) positive for Sca1 (rMC Sca1+ cells). Experimental design showing the AT2 cells (Alveolar type 2 cells) isolated from 35‐week‐old Sftpc^*CreERT2/+*^; *tdTomato* ^*flox*^ mice and gating strategy to sort Epcam+ and tdTomato+ (Tom^high^) cells. AT2 and rMC Sca1+ cells are co‐cultured in Matrigel for 14 days (alveolosphere assay). (b) Bright‐field and fluorescent images showing the formation of organoids from old‐AT2s/Tom^high^ (35‐week‐old mice) cells co‐cultured with rMC Sca1+ cells are isolated from Young‐C57BL/6 (8‐week‐old) mice, and such resident mesenchymal niche activity is lost in Old‐C57BL/6 (35‐week‐old) mice. (c) Quantification of colony forming efficiency confirming the loss in stem cell niche activity upon resident mesenchymal aging (*n* = 3/condition). (d) Experimental design showing the rMCs isolated either from saline or Bleomycin injured mice at day 14 post‐bleo injury from Old‐*Tg(Acta2‐CreERT2)/+*; *tdTomato* ^*flox*^ (52–56‐week‐old) female mice, and gating strategy to sort Cd31/Cd45/Epcam triple‐negative (rMC) positive for Sca1 (rMC Sca1+ cells). Experimental design showing the AT2 cells isolated from non‐injured Young‐C57BL/6 (8‐week‐old) mice and gating strategy to sort Epcam+ and Lysotracker+ (AT2) cells. AT2 and rMC Sca1+ cells are co‐cultured in Matrigel for 14 days (alveolosphere assay). (e) Bright‐field images showing organoids failed to form from saline or bleo‐injured mice (few cysts are formed in both conditions, which are around 50 μm in size). (f) Quantification of colony forming efficiency shows the loss in stem cell niche activity in saline or bleo‐injured mice upon resident mesenchymal aging. No significant changes are seen in the size (*n* = 3/condition). IP, Intraperitoneal. Scale bars: b, e‐ 100 μm. Statistical analysis was performed using (c, f)‐ unpaired two‐tailed *t*‐test. **p < 0.01; ***p < 0.001.

rMCs Sca1+ were co‐cultured with tdTom^high^‐AT2s from 35‐week‐old *Sftpc* ^*CreERT2/+*^; *tdTomato* ^*flox*^ mice. The gating strategy to sort rMCs Sca1+ and tdTom^high^‐AT2s is shown in Figure 1a. While young rMCs Sca1+ co‐cultured with mature AT2s in Matrigel for 14 days led to the formation of organoids with the expected colony forming efficiency (CFE) and size (Taghizadeh et al. 2021), rMCs Sca1+ from 35‐week‐old mice failed to display such niche activity (Figure 1b,c).

Given that the 35‐week‐old mesenchyme lost the niche activity, which we propose to be due to a progressive transition of the LIFs towards an aMYF‐like phenotype, we decided to use 52–56‐week‐old (aged) mice to investigate the process of fibrosis formation and resolution. 52–56‐week‐old *Tg(Acta2‐CreERT2)/+*; *tdTomato* ^*flox*^ female mice were intratracheally (IT) administered with saline (control mice) or bleomycin (bleo) at a dose which was reported to induce moderate fibrosis in young female mice (Lingampally et al. 2024). Tamoxifen was intraperitoneally injected (Tam‐IP) on days 5, 7, 9, and 11 to label the *Acta2*+ cells. Next, we sorted rMC Sca1+ cells from saline and bleo mice on day 14 using the gating strategy described in Figure 1d. AT2s from 8‐week‐old C57BL/6 mice were sorted as depicted in Figure 1d and co‐cultured with aged saline and bleo rMC Sca1+ cells in Matrigel for 14 days. Our results indicate that organoid formation is completely lost in aged saline mice (Figure 1e), confirming that the underlying cause for this result is the loss of the niche activity in the mesenchyme and not deficient AT2s, which in our conditions are coming from young mice and are therefore displaying robust AT2 stem cells. As previously described, bleo injury also causes loss of the niche activity (Figure 1e,f).

To summarize, these results indicate that in the alveolosphere model and our experimental conditions, mature AT2s are not the limiting factor for organoid formation; however, the niche activity displayed by rMCs Sca1+ is significantly impaired with aging.

### 3.2 Lineage Tracing of *Acta2+* Cells in Aged Healthy and Fibrotic Mouse Lungs

**FIGURE 2:**
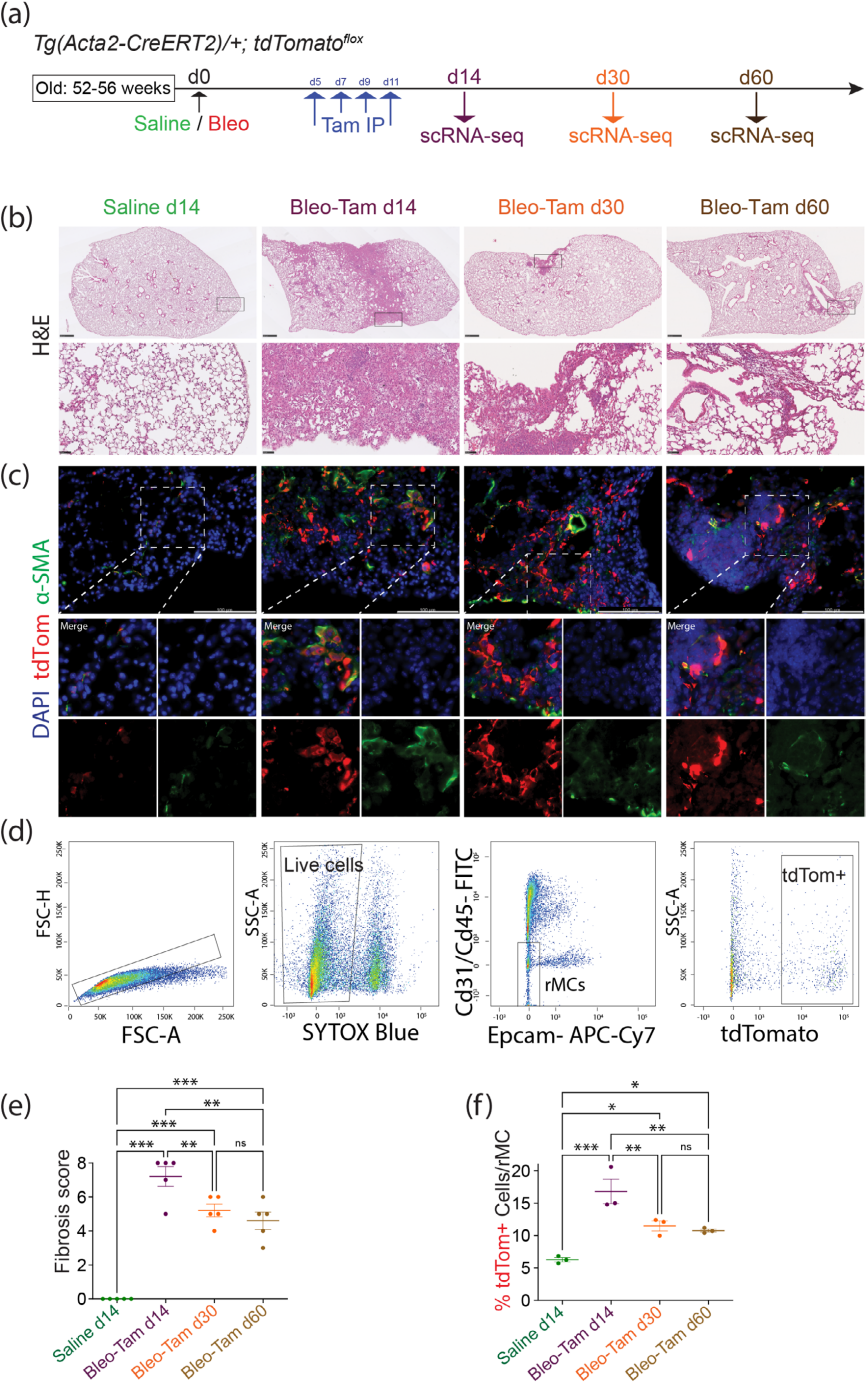
Lineage tracing of *Acta2*+ cells in saline and bleomycin‐injured mice. (a) 52–56‐week‐old female *Tg (Acta2‐CreERT2)/+*; *tdTomato* ^*flox/flox*^ mice are used to lineage label *Acta2*+ cells in saline, and bleo‐injured mice. Control lungs are collected at d14 following saline administration, and experimental lungs (Bleo‐Tam) are collected at d14, d30, and d60 following bleo injury. (b) Corresponding low and high magnification of H&E staining showing fibrosis formation at the Bleo‐Tam d14 peak of fibrosis and fibrotic lesions/regions are still present at resolution Bleo‐Tam d30 and Bleo‐Tam d60 following bleomycin injury. (c) IF staining against tdTom, α‐SMA, and DAPI shows tdTom/α‐SMA double‐positive cells in the alveolar region of saline lungs. Additionally, there is abundant recruitment of tdTom/α‐SMA double‐positive cells in the fibrotic regions of Bleo‐Tam d14, Bleo‐Tam d30, and Bleo‐Tam d60 lungs following bleo injury. (d) Gating strategy to sort SYTOX‐negative live lineage‐labeled tdTom+ cells. (e) Ashcroft score confirming massive formation in old mice upon bleo injury (b), saline d14 0 ± 0 score, Bleo‐Tam d14 7.2 ± 0.6 score, Bleo‐Tam d30 5.2 ± 0.4 score and Bleo‐Tam d60 4.6 ± 0.5 score. (f) Quantification of sorted tdTom+ cells over rMC (d) from saline d14 6.3% ± 0.3%, Bleo‐Tam d14 16.8% ± 1.9%, Bleo‐Tam d30 11.5% ± 0.8% and Bleo‐Tam d60 10.8% ± 0.2%. IP, Intraperitoneal. Scale bars: b: Low magnification‐ 500 μm, High magnification‐ 50 μm; c: 100 μm. Statistical analysis was performed using (e, f). One‐way ANOVA with Newman–Keuls *post hoc* test for multiple comparisons. *p < 0.05; **p < 0.01; ***p < 0.001.

52–56‐week‐old *Tg(Acta2‐CreERT2)/+*; *tdTom* ^*flox*^ female mice were intratracheally (IT) administered with saline (control mice) or bleo with 2 U.kg^−1^ body weight, a dose which was reported to induce moderate fibrosis in young female mice (Lingampally et al. 2024). Mice were injected with Tam‐IP post saline or after bleo administration to label *Acta2+* cells and examine their heterogeneity during fibrosis formation at d14 and their fate during resolution at d30 and 60 following bleo administration (Figure 2a). Experimental (bleo) and control (saline) mice were first examined on day (d) 14 at the peak of fibrosis.

Hematoxylin and Eosin staining showed abundant fibrotic areas upon bleo administration (Figure 2b). Furthermore, Bleo‐Tam d30 and Bleo‐Tam d60 lungs demonstrated the presence of significant residual fibrotic areas in the lung parenchyma (Figure 2b). Ashcroft scoring (Ashcroft, Simpson, and Timbrell 1988) at d14 was significantly increased in bleo compared to saline lungs confirming massive fibrosis formation. Ashcroft scoring at d30 and d60 indicated only partial fibrosis resolution (Figure 2e).

Next, we examined if aged mice compared to young mice (Lingampally et al. 2024) displayed, for the same bleo dose, increased fibrosis and delayed resolution by comparing their respective Ashcroft score. As expected, old mice displayed increased Ashcroft score both at d14 and at d60 (Figure S1) indicating elevated fibrosis formation and delayed fibrosis resolution compared to young mice as previously described.

Next, we examined the lineage‐traced *Acta2+* (tdTomato+) cells by immunofluorescence (IF) using antibodies against tdTomato (tdTom) and alpha‐smooth muscle actin (α‐SMA/Acta2). In saline conditions, we found tdTom/α‐SMA double‐positive cells in the parenchyma of the lung (Figure 2c). Next, we examined tdTom+ cells at the peak of fibrosis (d14) in bleo‐treated mice. We observed massive recruitment of tdTom/α‐SMA double‐positive cells in the fibrotic regions. These cells are still present in the lung both at d30 and d60 (Figure 2c).

Flow cytometry was used to sort and quantify the percentage of tdTom+ cells out of the Cd45/Cd31/Epcam triple‐negative resident mesenchymal cells (rMCs) in saline d14, Bleo‐Tam d14, Bleo‐Tam d30, and Bleo‐Tam d60 lungs. Representative FACS plots are shown in Figure 2d. SYTOX staining was used to exclude dead cells from the analysis and to sort live tdTom+ cells for scRNA‐seq experiments. FACS analysis confirmed a significant increase of tdTom+ cells over rMC in Bleo‐Tam d14 (16.8% ± 1.9%) compared to saline d14 (6.3% ± 0.3%). We also detected the significant presence of tdTom+ cells at Bleo‐Tam d30 (11.5% ± 0.8%) and Bleo‐Tam d60 (10.8% ± 0.2%) compared to saline d14 (Figure 2f).

### 3.3 *Acta2*+ Cells Lineage‐Labeled During Fibrosis Formation Massively Contribute to the *Cthrc1*+ aMYF Lineage

**FIGURE 3:**
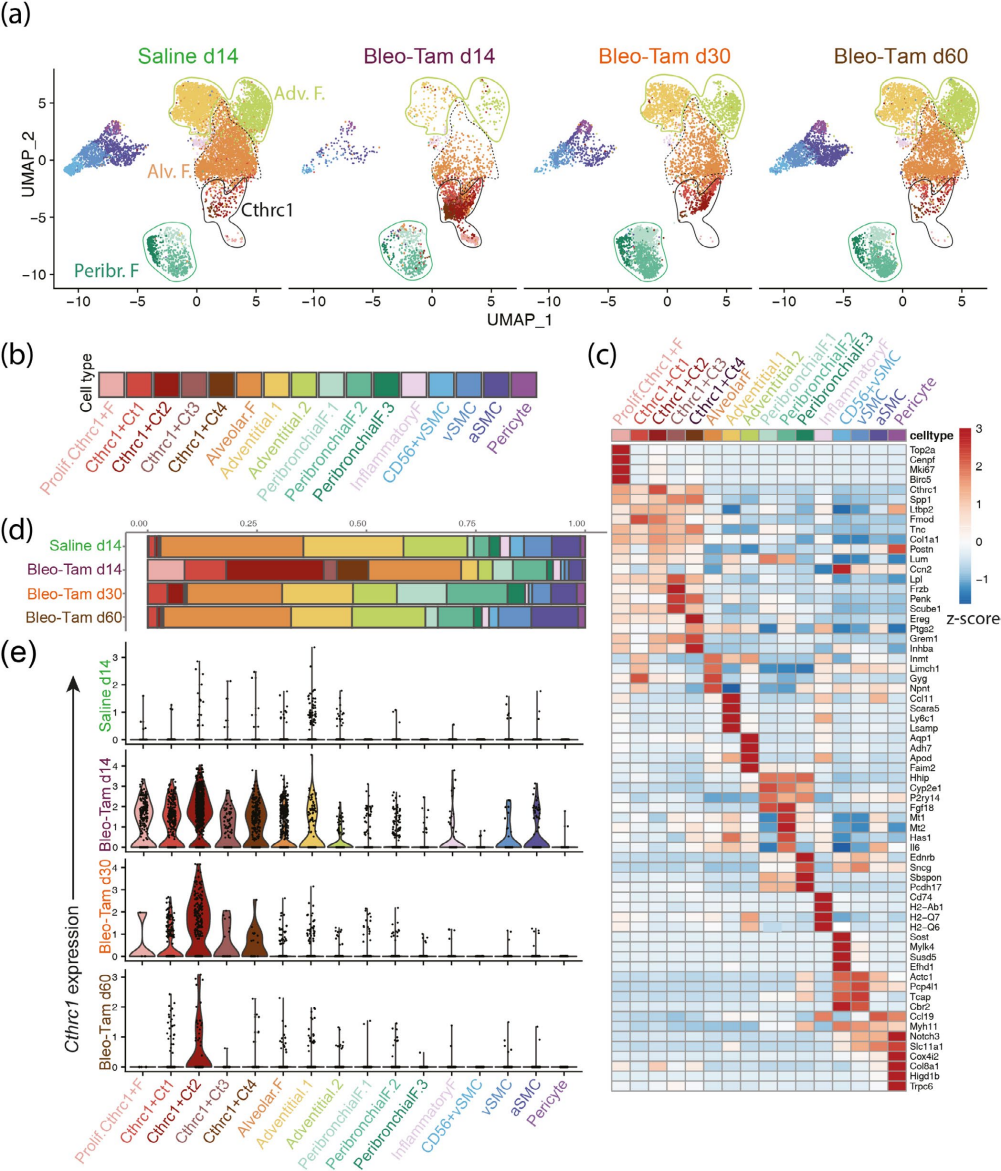
*Acta2+* cells contribute to multiple lineages in homeostasis and during fibrosis. (a) Integrated uniform manifold approximation and projection (UMAP) of *Acta2+* cells isolated from saline d14 and Bleo‐Tam d14, d30, and d60 lungs, showing 16 distinct clusters, including alveolar fibroblasts, peribronchial fibroblasts, adventitial fibroblasts and *Cthrc1+* myofibroblasts. (b) Nomenclature/cell identity of each cluster. (c) Heatmap of subpopulation markers. (d) Relative frequencies of each subpopulation in saline d14 and Bleo‐Tam d14, d30 and d60 lungs. Note that the *Cthrc1+* myofibroblasts are massively recruited (around 50%) in Bleo‐Tam d14 lungs, and proliferating *Cthrc1+* cells are also increased at the peak of fibrosis, suggesting aging is a predisposition for lung fibrosis. (e) Normalized expression of *Cthrc1* in the different subpopulations in saline d14 and Bleo‐Tam d14, d30 and d60 lungs. Note that the *Cthrc1* expression is high at the d14 peak of fibrosis. Expression was maintained at d30 and decreased during fibrosis resolution at d60. aSMC, airway smooth muscle cell; F, fibroblasts; prolif., proliferating; vSMC, vascular smooth muscle cell.

Lineage‐labeled *Acta2*+ (tdTom+) cells were sorted from saline d14 and Bleo‐Tam lungs at d14, d30, and d60 by negative gating for Cd31, Cd45 and Epcam (Figure 2d). Then, 9000 cells per condition were loaded for scRNA‐seq. After quality control (Figure S2 and Table 1), a total of 25,714 cells were recovered for the integrated analysis, 9088 cells from the saline d14, 3239 cells from Bleo‐Tam d14, 5700 cells from Bleo‐Tam d30 and 7687 cells from Bleo‐Tam d60. The integrated UMAP showed that tdTom+ cells contribute to 16 different subclusters including alveolar fibroblasts, adventitial fibroblasts (1,2), peribronchial fibroblasts (1–3), inflammatory fibroblasts, smooth muscle cells (SMC)/pericytes, proliferating and *Cthrc1+* aMYFs (Ct1‐4) (Figure 3a,b). Figure 3c shows the heatmap for the genes enriched for each cluster. The *Cthrc1+* aMYFs are enriched with fibrotic genes such as *Spp1*, *Ltbp2*, and *Col1a1*.

**TABLE 1:** Number of cells used for the integration of young and old mice samples.

Condition	Young mice (number of cells after QC)		Old mice (number of cells after QC)	
	Number of mice pooled before loading on 10×	Number of cells after QC	Number of mice pooled before loading on 10×	Number of cells after QC
Saline d14	3	2173	3	9088
Bleo‐Tam d14	1	6630	1	3239
Bleo‐Tam d30	NA	NA	2	5700
Bleo‐Tam d60	2	5029	1	7687
Total	6	13,832	7	25,714

The relative frequencies of the different clusters at each time point are shown in Figure 3d. The distribution of clusters supports our previous observations made in young mice (Lingampally et al. 2024). We observe that *Cthrc1+* aMYFs are increased upon bleo injury. Proliferating and *Cthrc1+* aMYFs (Ct1‐4) at d14 massively increased to represent around 50% of the tdTom+ cells analyzed in bleo vs. only 5% in saline d14.

The percentage of alveolar fibroblasts was also significantly decreased at d14 compared to saline. In contrast, at Bleo‐Tam d30, during the early fibrosis resolution phase, the proliferating and *Cthrc1+* aMYFs (Ct1‐4) were decreased (around 10%). The relative percentage of alveolar fibroblasts did not change. By late resolution, at Bleo‐Tam d60, the proliferating and *Cthrc1+* aMYFs (Ct1‐4) reached the basal level observed in saline level (around 5%). The alveolar fibroblasts showed an increase in their percentage at that time point. Violin plots indicated that proliferating and all *Cthrc1+* aMYF subclusters (Ct1‐4) express high levels of *Cthrc1*, with Ct2 showing the maximum expression. Indeed, high levels of *Cthrc1* expression were observed at the peak of fibrosis Bleo‐Tam d14, and the expression was maintained at Bleo‐Tam d30. Decreased *Cthrc1* expression in Bleo‐Tam d60 was observed during late resolution (Figure 3e). These results indicate that aged mice are more susceptible to fibrosis, as illustrated by an abundant *Cthrc1*+ population. In addition, we observed the maintenance of *Cthrc1* expression in Ct2 cells during fibrosis resolution at d60.

### 3.4 Deconvolution of the Heterogeneity of the *Cthrc1+* aMYFs and Their Fate During Fibrosis Formation and Resolution

**FIGURE 4:**
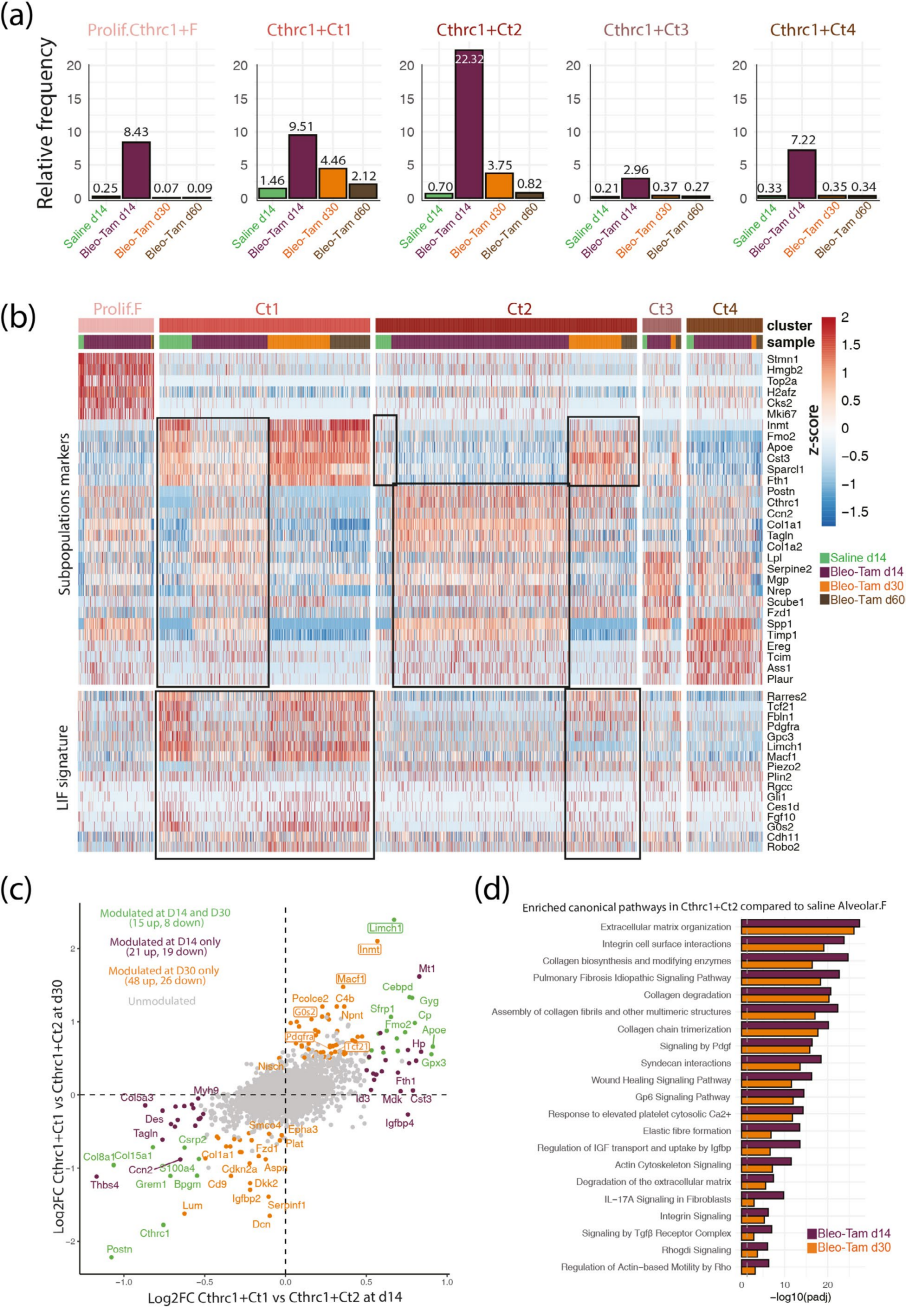
Comparison of *Cthrc1+* aMYFs during fibrosis formation and resolution. (a) *Acta2+* cells isolated from saline d14 and Bleo‐Tam d14, d30 and d60 lungs contributing to five clusters (Prolif.F, Ct1, Ct2, Ct3 and Ct4) of *Cthrc1+* cells. Relative frequencies of each *Cthrc1*‐expressing subpopulation at each time‐point. Note that the *Cthrc1+* myofibroblast cluster frequencies are high in Bleo‐Tam d14 lungs. (b) Heatmap showing the scaled expression of *Cthrc1*‐expressing subpopulation markers (top) and genes of the lipofibroblast (LIF) signature (bottom) in saline d14 and Bleo‐Tam d14, d30 and d60 lungs. (c) Correlation of the two Log2 Fold change obtained by comparing *Cthrc1+* Ct1 to Ct2 at d14 and d30 post‐bleo‐injury. Green dots correspond to genes significantly modulated (|log2FC| > 0.5 and *p*_adj_ < 0.05) in both time points. Purple and orange dots correspond to genes only significantly modulated at d14 or d30, respectively. Highlighted genes are canonical markers of LIF. (d) IPA canonical pathways enrichment was obtained from the differential analyses of Ct2 on d14 or d30 compared to saline d14 alveolar fibroblasts.

Next, we analyzed the *Cthrc1*+ subclusters from saline d14, Bleo‐Tam d14, d30, and d60 lungs (Figure 4). All *Cthrc1*+ subclusters were amplified in the context of fibrosis formation at d14. Indeed, the Ct2 (22.32%) cluster is most abundant over the other *Cthrc1*+ subclusters, such as Ct1 (9.51%), Ct3 (2.96%) and Ct4 (7.22%). However, at d30 and d60, during resolution, the relative percentages of *Cthrc1*+ (Ct1‐4) subclusters were significantly decreased (Figure 4a). Note that *Cthrc1+* aMYFs (Ct1‐4) at d14 massively increased from 25% in young mice (Lingampally et al. 2024) to 50% in old mice (Figure S3a,b,d). Interestingly, Ct2 is the most abundant cluster in both conditions, which shows the pivotal role of this population in fibrosis development.

Figure 4b shows the heatmap of the most expressed gene markers for each *Cthrc1+* subcluster. Ct1 contains *Inmt* and *Apoe*, two canonical alveolar fibroblast markers. The Ct1 cluster is significantly impacted, which leads to a decrease in its signature following bleo injury. At the peak of fibrosis, at d14, this cluster adopts an intermediate Ct2, Ct3, and Ct4 signature (shown in black box). Interestingly, during early (d30) and late fibrosis resolution (d60), the Ct1 signature returns to its native saline state, slightly increasing at d60. The Ct2 cluster in saline displayed a Ct1 signature. However, upon bleo injury, the Ct2 cluster loses the Ct1 signature and differentiates to a Ct2 status enriched with fibrotic genes such as *Postn*, *Col1a1*, *Cthrc1*, and *Tagln*. This cluster also adopts an intermediate Ct3 and Ct4 signature (shown in black box). Interestingly, during early (d30) and late (d60) fibrosis resolution, the Ct2 signature returns to its native saline/Ct1 status. Ct4 and Ct3 clusters showed a similar trend to Ct1 and Ct2 during fibrosis formation at d14 and resolution at d60. However, the shift towards the Ct1 signature is not seen due to the low number of cells during resolution.

Next, when we interrogated the previously described LIF signature (Lingampally et al. 2024) into *Cthrc1+* (Ct1‐4) subclusters during fibrosis formation and resolution, Ct1 shows the highest level of all clusters. The Ct1 cluster during the peak of fibrosis d14 displayed a decrease in the LIF signature compared to saline. However, during resolution at d30 and d60, the LIF signature is enriched in the Ct1 cluster. Similarly, the Ct2 cluster in saline displays a high LIF signature. Upon bleo injury, the signature decreases at d14 and recovers during resolution to its saline level.

Correlation analysis comparing *Cthrc1+* Ct1 to Ct2 at d14 and d30 also confirms that the expression of canonical markers of LIFs such as *Limch1*, *Inmt*, *Macf1*, *Apoe*, and *Tcf21* are upregulated at d30 (Figure 4c), therefore supporting a MYF to LIF transition.

IPA canonical pathways enrichment analysis of Ct2 at d14 and d30 compared to saline d14 alveolar fibroblasts showed the enrichment of pathways like Pdgf, Gp6, Tgfβ, and IL‐17A at d14, and their subsequent downregulation at d30 (Figure 4d).

To decipher the effects of aging on *Cthrc1+* Ct1 and Ct2 cells, we have performed a correlation analysis comparing *Cthrc1+* Ct1 to Ct2 at d14 in young and old mice. The Ct1+ cells (from both young and old mice) are enriched with LIF markers such as *Limch1* and *Apoe* with low levels of expression of fibrotic markers such as *Cthrc1*, *Postn*, and *Tagln* (Figure S4a).

Likewise, IPA canonical pathways enrichment analysis of *Cthrc1+* Ct1 to Ct2 cells at d14 reveals a similarity in the regulation of fibrotic pathways between young and old mice (Figure S4b).

### 3.5 Characterization of *Cthrc1+* aMYF Subclusters in Saline and Fibrotic Lungs

To verify the heterogeneity of *Cthrc1+* aMYFs (Ct1‐4) (Figure 4b), we have performed the RNAscope assay combined with immunofluorescence (IF).

**FIGURE 5:**
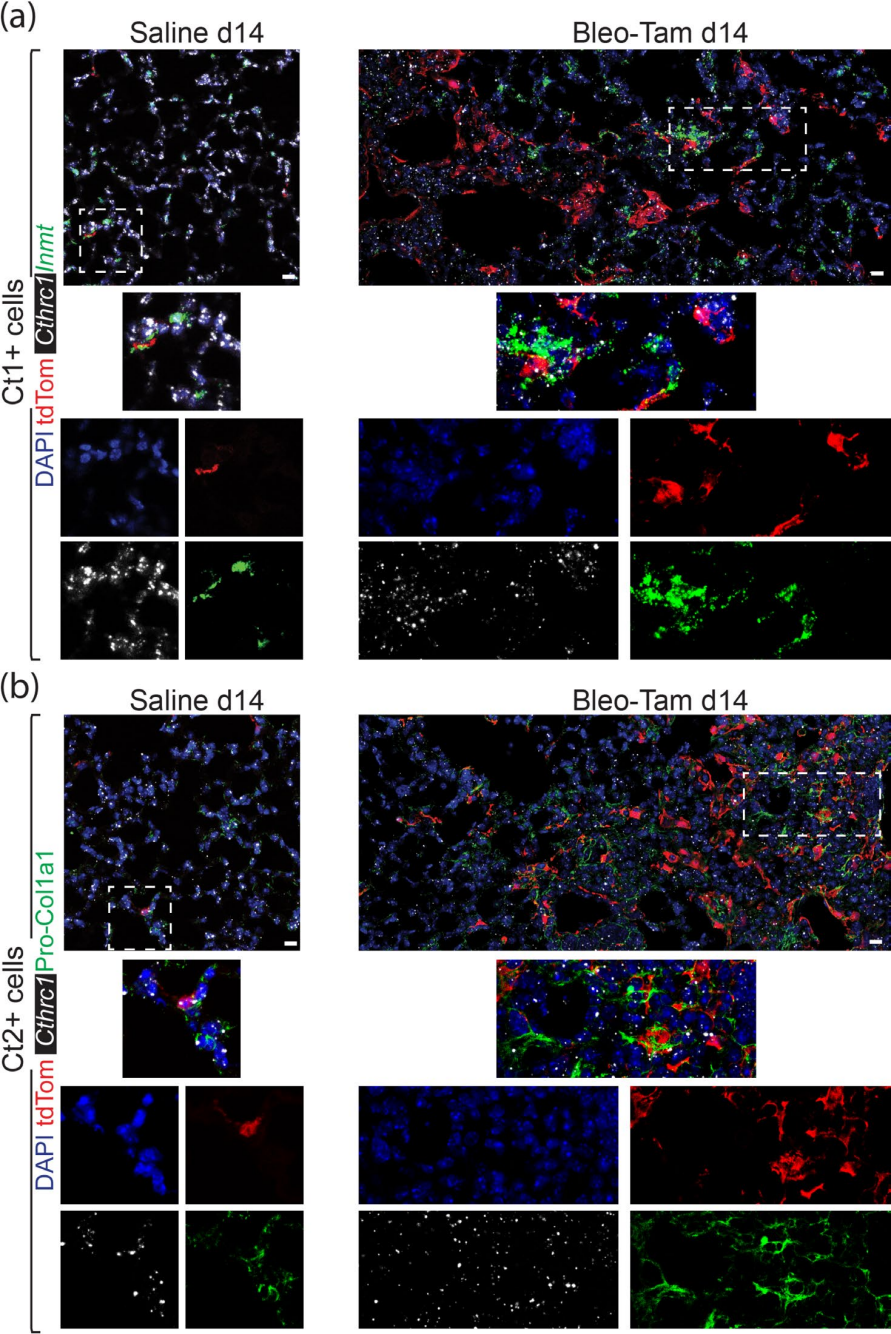
Characterization of Ct1 and Ct2 cells in saline and Bleo‐Tam d14 lungs. (a) RNA‐scope of *Cthrc1+* aMYF marker *Cthrc1* and Ct1‐cluster marker *Inmt* showing colocalization with tdTom+ cells. (b) RNA‐scope of *Cthrc1+* aMYF marker *Cthrc1* combined with immunofluorescence (IF) for Pro‐Col1a1 (Ct2‐cluster marker), showing colocalization with tdTom+ cells. Scale bars: (a, b): 150 μm.

First, we identified Ct1 and Ct2 cells in saline lungs (Figure 5). In the Bleo‐Tam d14 lungs, we observed tdTom/*Cthrc1*/*Inmt* triple‐positive Ct1 cells and tdTom/*Cthrc1* double‐positive aMYFs (Figure 5a). Likewise, we observed a high abundance of tdTom/*Cthrc1*/ Pro‐Col1a1 triple‐positive Ct2 cells and tdTom/*Cthrc1* double‐positive aMYFs (Figure 5b).

Conversely, we observed the absence of Ct3 and Ct4 cells in saline lungs (Figure S5). However, the fibrotic areas of the Bleo‐Tam d14 lungs contained tdTom/*Cthrc1* double‐positive aMYFs and tdTom/*Cthrc1*/*Serpine2* triple‐positive Ct3 cells (Figure S5a). We also found tdTom/*Cthrc1*/*Timp1* triple‐positive Ct4 cells and tdTom/*Cthrc1* double‐positive aMYFs (Figure S5b).

### 3.6 Comparison of Young Versus Old Alveolar Fibroblast Subpopulations During Fibrosis Formation and Resolution

**FIGURE 6:**
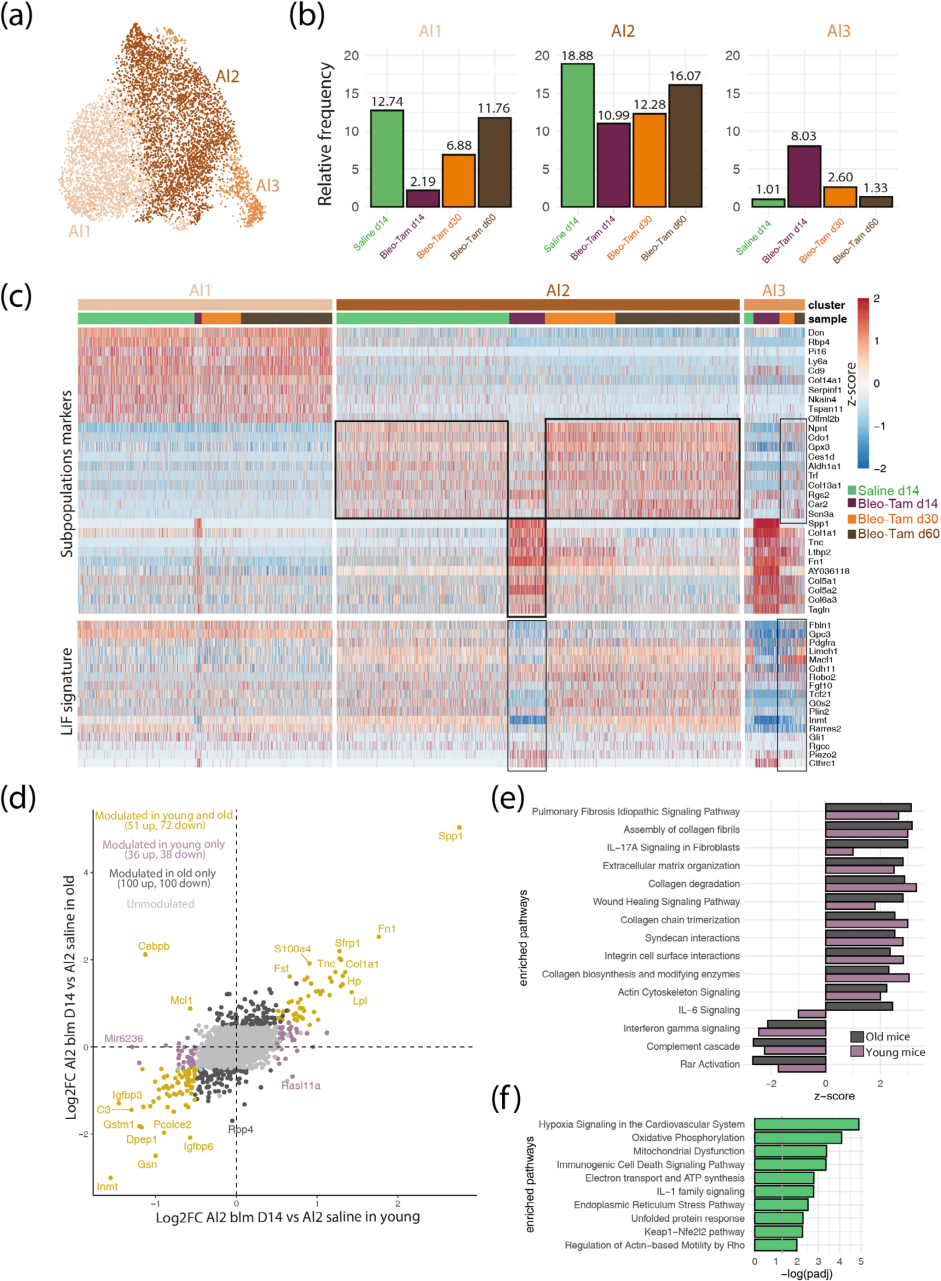
Comparison of alveolar fibroblast subpopulations during fibrosis formation and resolution in old mice. (a) Uniform manifold approximation and projection (UMAP) of *Acta2+* cells isolated from saline d14 and Bleo‐Tam d14, d30 and d60 lungs contributing to three alveolar fibroblast subpopulations (Al1, Al2, and Al3). (b) The relative frequencies of each subpopulation at each time‐point. (c) Heatmap showing the scaled expression of alveolar fibroblast subpopulation markers (top) and genes of the lipofibroblast (LIF) signature (bottom) in saline d14 and Bleo‐Tam d14, d30 and d60 lungs. (d) Correlation of the two log2 Fold change obtained by comparing Al2 of the Bleo‐Tam d14 sample to Al2 of the saline d14 sample from old (52–56‐week‐old) and young (8–12‐week‐old) mice. Golden dots correspond to genes significantly modulated (|log2FC| > 0.5 and *p*_adj_ < 0.05) in both old and young. Dark gray and mauve dots correspond to genes that are only significantly modulated in old or young, respectively. (e) IPA Canonical Pathways enrichment was obtained from the differential analyses of Al2 of the Bleo‐Tam d14 sample compared to Al2 of the saline d14 sample in both the old and young mice. (f) IPA canonical pathways enrichment obtained from the upregulated genes in Al2 of saline d14 sample from old mice compared to Al2 of saline sample from young mice.

Next, we carried out a sub‐clustering of the alveolar fibroblasts (Al). As in our previous study, alveolar fibroblasts can be subdivided into Al1, Al2, and Al3 (Figure 6a). Figure 6b shows the relative frequencies of the respective sub‐clusters; Al2 is the abundant cluster of the alveolar fibroblasts. Interestingly, at the peak of fibrosis (d14), Al1 and Al2 decrease in proportions, which was partially rescued during resolution at d30 and d60. In contrast, the Al3 proportion increased at d14 and conversely decreased during resolution. These relative frequency changes for these clusters were not significantly observed in young mice (Figure S3c,d). We also did not observe a higher proportion of Al3 in old mice compared to young in saline d14 samples (Figure S3c).

Figure 6c shows the heatmap of the most expressed gene markers for each alveolar fibroblast sub‐cluster. At d14, following bleo injury and at the peak of fibrosis, the Al2 cluster shows a decrease in its native signature. It acquires the expression of fibrotic genes (*Spp1*, *Col1a1*, *Tnc, Ltbp2*, and *Tagln*), adopting Al3 characteristics. In contrast, during resolution, Al2 cells return to the native/saline state by losing the expression of the fibrotic genes. At the peak of fibrosis d14, the LIF signature in Al2 cluster decreases, which is recovered during fibrosis resolution. Al1 cluster also show a similar trend (acquisition of Al3 characteristics and decreased LIF signature following bleo at d14).

The Al3 cluster, quantitatively enriched following bleo at d14, displays high‐level expression of fibrotic genes (*Spp1*, *Col1a1*, *Tnc, Ltbp2*, and *Tagln*). In young mice, our initial analysis indicated that Al3 cells are primarily observed in Bleo‐Tam d14 but not in Tam‐Bleo d14. This suggests that these cells were initially *Acta2*‐. However, during fibrosis formation, they acquire *Acta2* expression and are captured by our lineage tracing approach as *Acta2*+ cells. Based on the LIF signature heatmaps and the trajectory analysis, we proposed that these cells differentiate towards *Cthrc1*+ Ct1 cells (Lingampally et al. 2024).

During resolution, the Al3 cluster decreases its fibrotic signature and shifts towards an Al2 phenotype. Similarly, the decreased LIF signature in this cluster at d14 is also rescued during fibrosis resolution d30 and d60 (Figure 6c).

Therefore, it is likely that Al2 *Acta2*‐ cells are a primary source for the Al3 *Acta2*+ cluster following bleo injury. Correlation analysis comparing Al2 clusters of Tam‐Bleo d14 to saline d14 in young and old mice shows the activation of Al2 cluster with fibrotic genes (*Spp1*, *Sfrp1*, *Col1a1*, *Tnc* and *Fn1*) (Figure 6d).

At the transcriptomic level and in our experimental conditions, direct comparison between old and young samples was challenging because of the differences in the UMI content between the two sets of experiments (Figure S3e). Nevertheless, an indirect comparison can be performed in fibrotic conditions by confronting the log_2_FC in Al2 between Bleo‐d14 to saline d14 in old and young mice (Figure 6d).

Overall, IPA canonical pathways enrichment analysis indicated some age‐dependent modulations (Figure 6e). Interestingly, IL‐17A and IL‐6 signaling were upregulated in Al2 old versus young (Figure 6e). To check for age‐related differences in saline d14, we focused on upregulated genes in old compared to young Al2 cells. The genes upregulated in young versus old Al2 cells were not analyzed due to the lower sequencing depth in old mice, resulting in a limited number of detected genes. Our analysis indicated the enrichment of several pathways in Al2 cells, such as hypoxia signaling, oxidative phosphorylation, IL‐1 signaling, and regulation of Actin‐based motility by Rho, which could be relevant for the defective resolution in old mice (Figure 6f).

### 3.7 Comparison of LIFs (Al2 Cells) During Fibrosis Formation (d14) to Fibrosis Resolution (d60) in Young and Old Mice

While the lineage tracing model has limitations, our data indicate that we can efficiently capture a large variety of fibroblasts, including several pathologic subpopulations mainly detected in bleo‐treated mice. The possibility that part of the *Cthrc1+* cells undergo apoptosis is an important aspect to consider. We have investigated this hypothesis using two approaches.

First, we performed a TUNEL assay (Figure S6) to explore the possibility that some of the *Cthrc1*+ aMYFs undergo apoptosis. Our results indicate that a small fraction of tdTom+ cells turned up TUNEL+ at the peak fibrosis (d14). Interestingly, we also observed both apoptotic tdTom+ and tdTom− cells during early fibrosis resolution (at d30). Whether apoptotic tdTom+ cells belong to a specific Ct subcluster is still unclear and should be considered when interpreting our lineage‐tracing results. However, no apoptotic cells within the tdTom+ cell population could be detected during late fibrosis resolution (d60).

**FIGURE 7:**
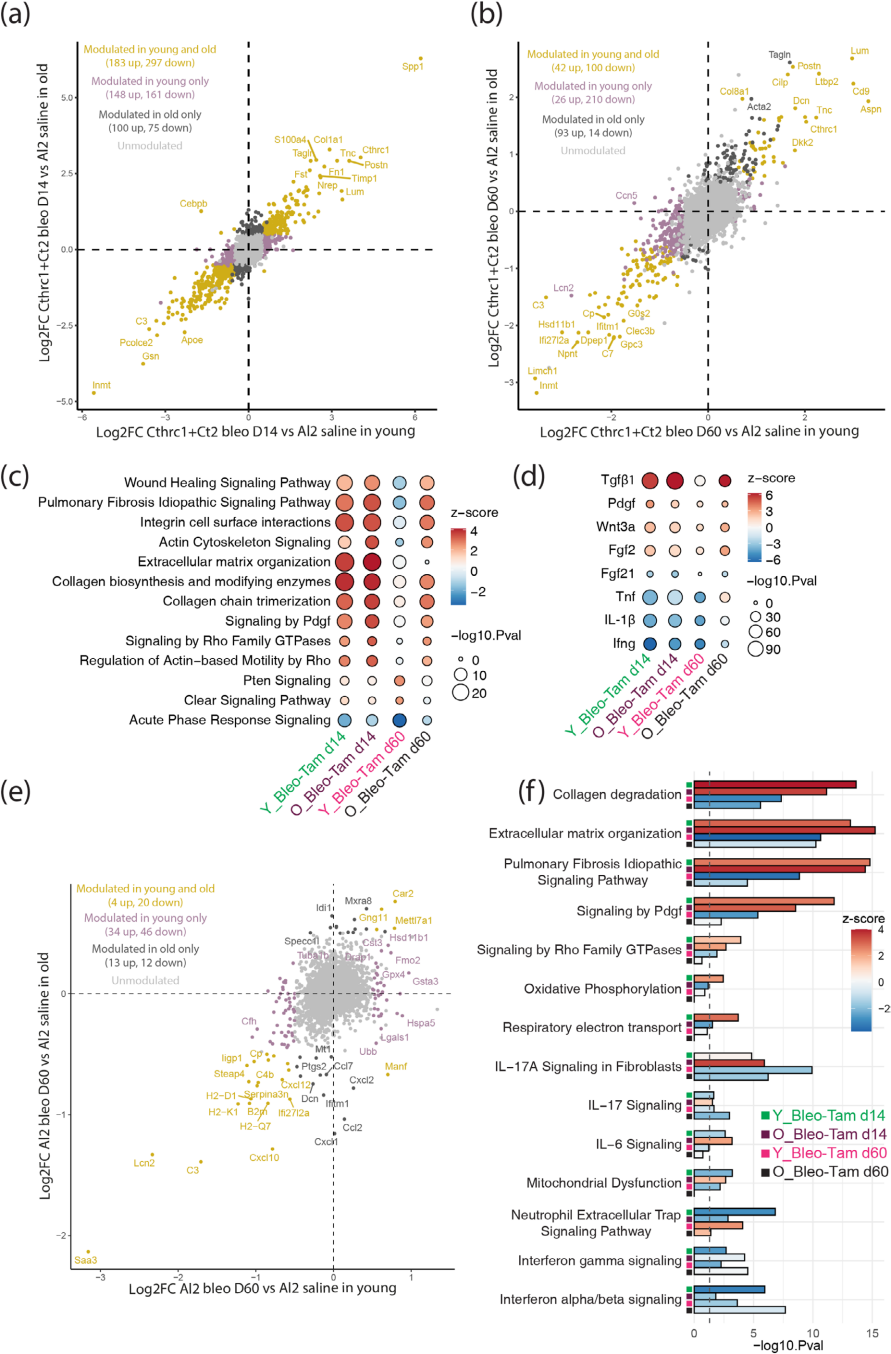
Comparison of aMYF versus LIF (*Cthrc1+* Ct2 vs. Al2 cells) during fibrosis formation (d14) and resolution (d60) in young and old mice. (a) Correlation of the two log2 Fold change obtained by comparing *Cthrc1+* Ct2 Bleo‐Tam d14 to Al2 saline from young and old mice. Golden dots correspond to genes significantly modulated (|log2FC| > 0.5 and *p*_adj_ < 0.05) in both young and old. Mauve and dark gray dots correspond to genes only significantly modulated in young or in old mice, respectively. (b) Correlation of the two log2 Fold change obtained by comparing *Cthrc1+* Ct2 Bleo‐Tam d60 to Al2 saline from young and old mice. Golden dots correspond to genes significantly modulated (|log2FC| > 0.5 and *p*_adj_ < 0.05) in both young and old. Mauve and dark gray dots correspond to genes only significantly modulated in young or old mice, respectively. (c, d) Integrated IPA canonical pathways (c) and Growth factors/cytokines (d) enrichment obtained from the differential analyses of: *Cthrc1+* Ct2 Bleo‐Tam d14 compared to Al2 saline and *Cthrc1+* Ct2 Bleo‐Tam d60 compared to Al2 saline in both young and old mice. (e) Correlation of the two log2 Fold change obtained by comparing Al2 Bleo‐Tam d60 to Al2 saline from young and old mice. Golden dots correspond to genes significantly modulated (|log2FC| > 0.5 and *p*_adj_ < 0.05) in both young and old. Mauve and dark gray dots correspond to genes only significantly modulated in young or in old mice, respectively. (f) Integrated IPA canonical pathways enrichment obtained from the differential analyses of Al2 (Bleo‐Tam d14) and Al2 (Bleo‐Tam d60) compared to Al2 saline in both young and old mice. O, old mice; Y, young mice.

Next, we analyzed the potential link with cell death within the DE gene signature comparing the most activated Ct2+ aMYF at the fibrosis peak (Bleo‐Tam d14) (Figure 7a) and at the end of resolution (Bleo‐Tam d60) (Figure 7b) to the LIF alveolar Al2 saline cells from either young or old mice. IPA canonical pathways enrichment analysis did not show any clear prediction associated with an induction of cell death, with an inhibition of ferroptosis and immunogenic cell death pathways in both young and old mice. Overall, our data indicate that specific fibroblasts populations do not undergo extensive cell death during fibrosis resolution. This strongly suggests that a large proportion of these cells are still viable and, according to our analysis, have the potential to transition back to their native cellular state to restore lung homeostasis.

At both d14 and d60, we observed upregulation of fibrotic genes (*Spp1*, *Col1a1*, *Ltbp2*, *Postn*, *Cthrc1*, and *Tagln*) and downregulation of LIF markers (*Inmt*, *Limch1*, *G0s2*, and *Apoe*) in Ct2 versus Al2 cells in young and old mice (Figure 7a,b). Interestingly, at d60, old mice Ct2 versus Al2 cells showed an upregulation of *Acta2* and *Tagln*, suggesting a persistent/delayed fibrosis resolution supported by the presence of tdTom/α‐SMA double‐positive cells in Bleo‐Tam d60 lungs in Figure 2c. Furthermore, IPA canonical pathways enrichment analysis indicated delayed fibrosis resolution in old versus young mice at d60 with the maintenance of IPF signaling, integrin cell surface interaction, actin cytoskeleton signaling, collagen biosynthesis and trimerization, and Pdgf signaling (Figure 7c). Growth factors/cytokines enrichment analysis indicated the persistence of Tgfβ1, Wnt3a, Fgf2, and Tnf in Ct2 cells in old versus young at d60 (Figure 7d).

Next, we compared Al2 Bleo‐Tam d60 to Al2 saline cells from young and old mice. Interestingly, upregulation of the LIF marker *Fmo2* was observed only in young mice while old mice showed downregulation of inflammatory modulators (*Ccl2*, *Cxcl2*, and *Cxcl1*). Additionally, IPA canonical pathways enrichment analysis indicated maintenance of ECM organization, IPF, Pdgf, interferon alpha/beta and gamma signaling in old versus young LIFs/Al2 cells at d60 (Figure 7e,f), indicating persistence of fibrosis in old mice.

## 4 Discussion

IPF is a lethal lung disease caused by irreversible fibrosis and associated with aging. Considering these later criteria, it has been proposed that old mice rather than young mice represent a much more relevant model to study IPF due to elevated fibrosis and delayed resolution following bleo exposure (Caporarello et al. 2020; Jenkins et al. 2017; Redente et al. 2011).

The underlying causes for such differences in the response to bleomycin‐induced fibrosis in old versus young mice are progressively emerging. Among them, others and we have proposed that the nature of the lung mesenchyme is changing. Our study provides evidence for a LIF‐to‐aMYF‐like differentiation process during aging. The alveolosphere assay has been instrumental to evaluate the activity of the resident mesenchymal cells in supporting the proliferation of AT2 stem cells. Using this model, we show that this niche activity is already lost in 8‐month‐old mice, serving as a rationale to use 12‐month‐old mice for our studies instead of the 18–24‐month‐old mice currently used for studies involving aging. In 12‐month‐old mice, we observed increased fibrosis formation and delayed fibrosis resolution compared to 2‐month‐old mice for the same dose of bleo.

Interestingly, we previously reported that the rMC Sca1+ niche activity was also impacted by obesity and gender, with *ob/ob* versus wild type (WT) mice and male versus female WT mice displaying lower niche activity (Taghizadeh et al. 2021). These observations made in mice are consistent with what is observed in humans, where COVID‐19‐infected older men suffering from metabolic diseases are more prone to develop pneumonia (Cai et al. 2020; Muniyappa and Gubbi 2020; Nouri‐Keshtkar et al. 2021; Zhang et al. 2020).

Recently, the analysis of the pathways at the transcriptomic and proteomic level associated with fibrosis appears to be similarly regulated between young and aged mice (Klee et al. 2023). A similar observation was done by analyzing the metabolomic and lipidomic changes in old versus young mice following bleo injury (Weckerle et al. 2022).

**FIGURE 8:**
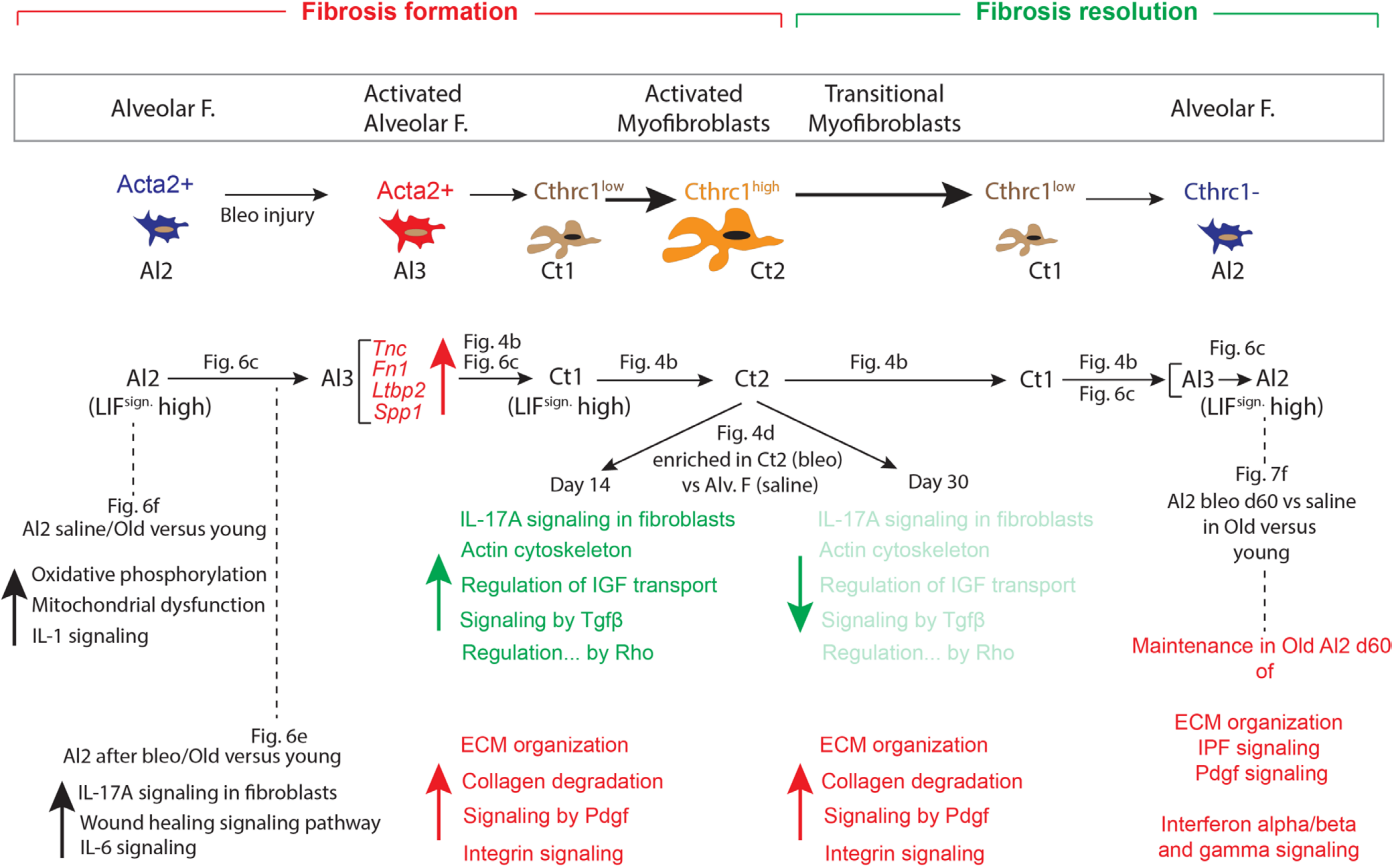
Model of LIF‐to‐aMYF reversible switch during fibrosis formation and resolution in old mice. The main contributors during fibrosis formation to *Cthrc1*+ aMYF (Ct1‐Ct4) are *Acta2*+ LIF^high^ alveolar fibroblasts (Al2), which get activated and differentiate to Al3 to express a fibrotic signature (*Spp1*, *Tnc*, *Ltbp2* and *Fn1*). These activated Al3 cells eventually differentiate into *Cthrc1* ^Low^ LIF^high^ aMYFs belonging to the Ct1 cluster. These Ct1 cells further differentiate massively to contribute to Ct2 *Cthrc1* ^high^ aMYFs. During fibrosis resolution, the opposite occurs with Ct2 cells decreasing their signature and differentiating into Ct1 cells, which eventually differentiate back to the Al3 cluster and finally to the *Cthrc1*‐ LIF^high^ Al2 alveolar fibroblasts cluster. The pathways modulated in Al2 saline (old vs. young), Al2 after bleo at d14 (old vs. young), and enriched in Ct2 (bleo) versus Alv. F (saline) are shown. Please note that the maintenance in Al2 d60 versus saline (old vs. young) of ECM organization, IPF, Pdgf, and interferon signaling indicate delayed fibrosis resolution in old mice. IL, interleukin; Sign, signature; Green, pathways upregulated in Ct2 during fibrosis formation. Note that some of these pathways are also upregulated during resolution, albeit at a lower level (lighter shade of green). Red: Pathways specifically upregulated during fibrosis resolution.

However, in old mice, an increased inflammatory state was detected (Klee et al. 2023). Interestingly, we noticed a persistence of IL‐1, IL‐17A, and IL‐6 signaling in old Al2 cells in saline and after bleo injury (please see the model in Figure 8), supporting a dysfunctional regulation of the immune response in old mice. Further studies will have to be done to understand the function of the immune response in the context of aging and fibrosis formation.

We previously proposed that Al3 differentiate towards *Cthrc1*+ Ct1 cells (Lingampally et al. 2024). In the future, lineage tracing using Cre/Dre dual recombinase to target Al3 should be carried out to demonstrate that Al3 cells differentiate into *Cthrc1*+ Ct1 cells.

Our lineage tracing of the aMYFs indicates that the basic cellular mechanisms are conserved between old and young mice. Our results suggest an alveolar fibroblast to a *Cthrc1*+ aMYF reversible switch during fibrosis formation and resolution (see model in Figure 8). Interestingly, we identified pathways downregulated in *Cthrc1*+ aMYF (cluster Ct2) between Bleo‐Tam d30 and Bleo‐Tam d14. These pathways include IL‐17A and signaling by Tgfβ, which are major contributors to fibrosis formation. Such downregulation over time indicates that the resolution process occurs in old mice. However, we also found other signaling pathways, such as signaling by Pdgf, integrin signaling, and collagen degradation, which are still highly active during resolution (d30 and d60) and consistent with the observed persistence of fibrosis.

Interestingly, we have previously reported that metformin, an anti‐diabetic drug also thought to have an impact on longevity (Barzilai et al. 2016), is capable of triggering aMYF‐to‐LIF differentiation in vitro (Kheirollahi et al. 2019; Vasquez‐Pacheco et al. 2024) and improve fibrosis resolution in vivo in mice (Kheirollahi et al. 2019; Rangarajan et al. 2018). The clinical use of metformin in combination with the gold standard treatments (pirfenidone or nintedanib) to attenuate fibrosis is still in its infancy. Still, it highlights how enhancing the aMYF‐to‐LIF differentiation process could be used clinically to provide new treatments for IPF patients. In the future, new drugs capable of triggering LIF differentiation, with or without an impact on the fibrotic profile, are likely to be instrumental in restoring normal lung function and could be identified using our recently described in vitro model using WI‐38 human embryonic lung fibroblasts (Vasquez‐Pacheco et al. 2024).

In conclusion, our data from old mice support the results obtained from young mice concerning the LIF‐to‐aMYF reversible switch. We propose that functionally targeting this switch by increasing LIF differentiation and/or decreasing aMYF differentiation will be a promising approach for innovative IPF treatments. The relevance for fibrosis development and resolution of the differentiation status of the alveolar fibroblast Al2 in old versus young mice before and after injury will require further studies. The essential pathways activated in the Ct2 cluster during fibrosis formation and resolution identified in our studies in young and old mice will be a starting point to identify potential therapeutic drugs.

## Author Contributions

Marin Truchi carried out the bioinformatic analysis on the generated mouse samples. Xianrong Shi, Yuqing Zhou, Xuran Chu, Stefan Hadzic, Esmeralda Vasquez‐Pacheco, and Georgios‐Dimitrios Panagiotidis helped with the bleo injury model. Janine Koepke and Ana Ivonne Vazquez‐Armendariz performed the FACS and scRNA‐seq analysis. Hector A. Cabrera‐Fuentes, Susanne Herold, Christos Samakovlis, Jin‐San Zhang, Werner Seeger, and Elie El Agha contributed to the project's conceptualization. Arun Lingampally, Bernard Mari, Saverio Bellusci, and Chengshui Chen conceptualized the project, provided funding, analyzed and interpreted the data, and wrote the manuscript. All authors read and agreed with the results presented in the manuscript.

## Conflicts of Interest

The authors declare no conflicts of interest.

## Supporting information


Data S1.

## Data Availability

The data that support the findings of this study are openly available in gene expression omnibus (GEO) repository at https://www.ncbi.nlm.nih.gov/geo/, reference number GSE253453, GSE221402, GSE223664.
