# Supplementary material for: Unraveling Alveolar Fibroblast and Activated Myofibroblast Heterogeneity and Differentiation Trajectories During Lung Fibrosis Development and Resolution in Young and Old Mice
Source: Aging Cell. 2025 Feb 13;24(5):e14503. doi: 10.1111/acel.14503 (PMC12073919; doi:10.1111/acel.14503)
Supplement: Supplementary file 1 — Data S1. [file ACEL-24-e14503-s001.pdf]

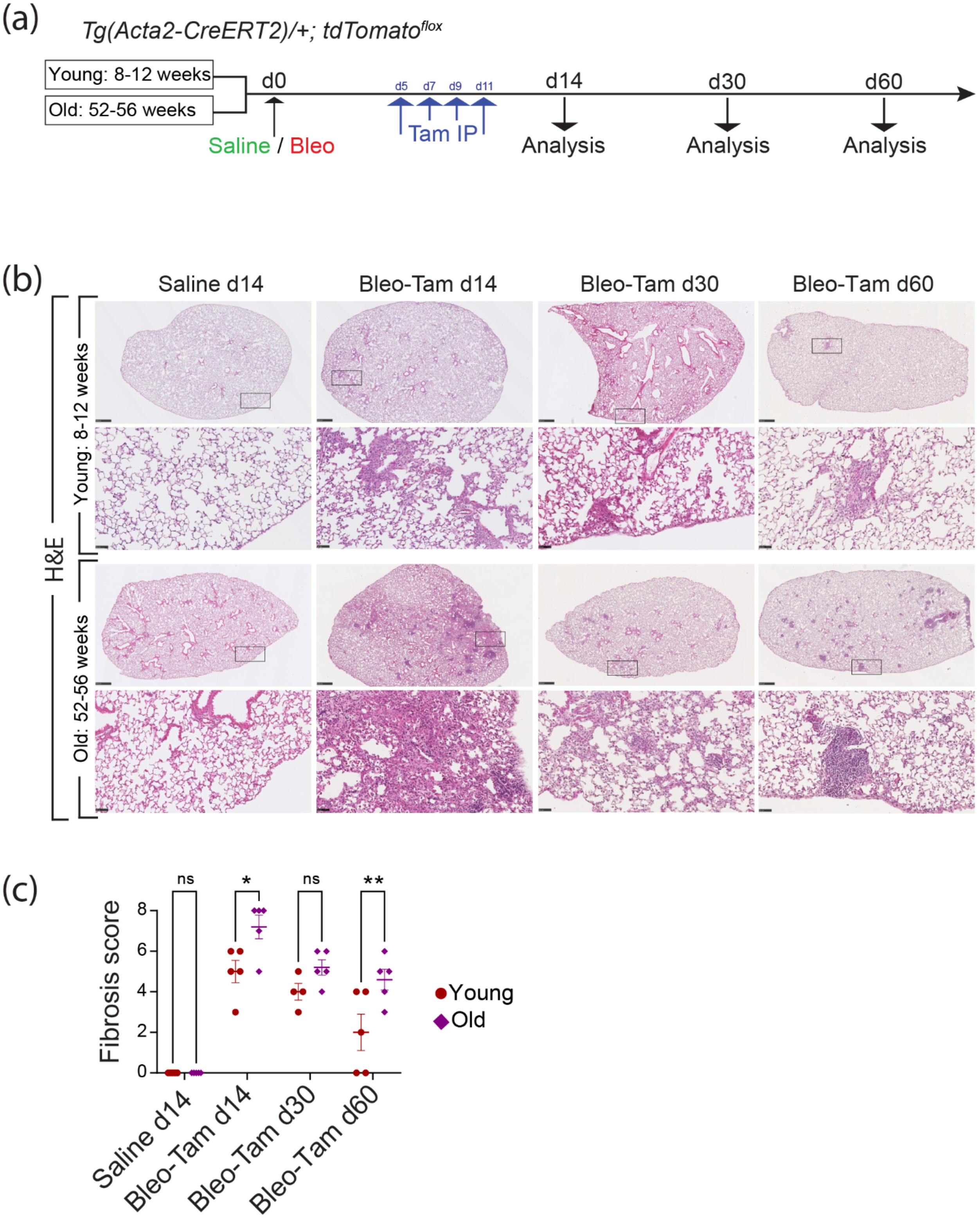

(a)
Tg(Acta2-CreERT2)/+; tdTomato^flox
Young: 8-12 weeks
Old: 52-56 weeks
d0
d5 d7 d9 d11
d14
d30
d60
Saline / Bleo
Tam IP
Analysis
Analysis
Analysis
(b)
Saline d14
Bleo-Tam d14
Bleo-Tam d30
Bleo-Tam d60
H&E
Young: 8-12 weeks
Old: 52-56 weeks
(c)
Fibrosis score
ns
*
ns
**
Young
Old
Saline d14
Bleo-Tam d14
Bleo-Tam d30
Bleo-Tam d60

Figure S1**: Comparison of fibrosis in young versus old mice. (a)** Female *Tg(Acta2-CreERT2)/+;tdTomato^{flox}* young (8-12-week-old) and old (52-56-week-old) mice are either subjected to saline or 2 $U.kg^{-1}$ bleomycin (bleo). Control lungs are collected at d14 following saline administration, and experimental lungs (Bleo-Tam) are collected at d14, d30, and d60 following bleomycin injury. **(b)** Corresponding low and high magnification of H&E staining showing massive fibrosis formation in old mice lungs compared to young at Bleo-Tam d14. Additionally, fibrotic lesions/regions are still present at resolution Bleo-Tam d30 and Bleo-Tam d60 in young and old mice lungs. **(c)** Comparison of the fibrotic scores between young and old mice at different time points and conditions. Scale bars: b: Low magnification- 500 μm, High magnification- 50 μm; Statistical analysis was performed using (c) 2-way ANOVA with Šídák's *post hoc* test for multiple comparisons. *p<0.05; **p<0.01.

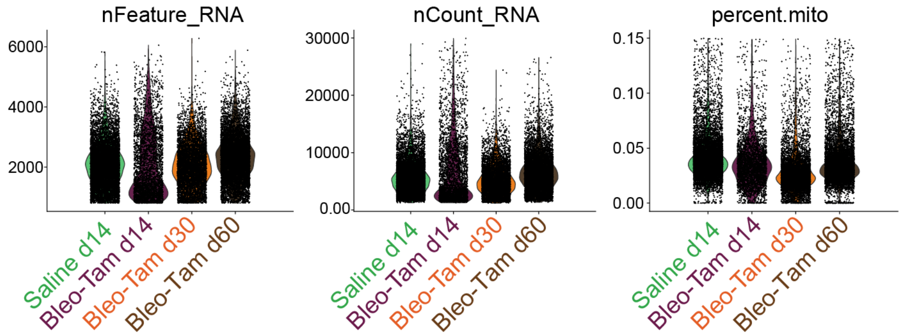

Figure S2**: Quality control of scRNA-seq datasets from lineage-labeled *Acta2*+ (tdTom+) cells sorted from saline d14 and Bleo-Tam lungs at d14, d30 and d60 (52-56-week-old mice).** nFeature_RNA (the number of genes detected in each cell), nCount_RNA (the total number of RNA molecules detected within a cell), and the percentage of mitochondrial genes are quantified for each condition.

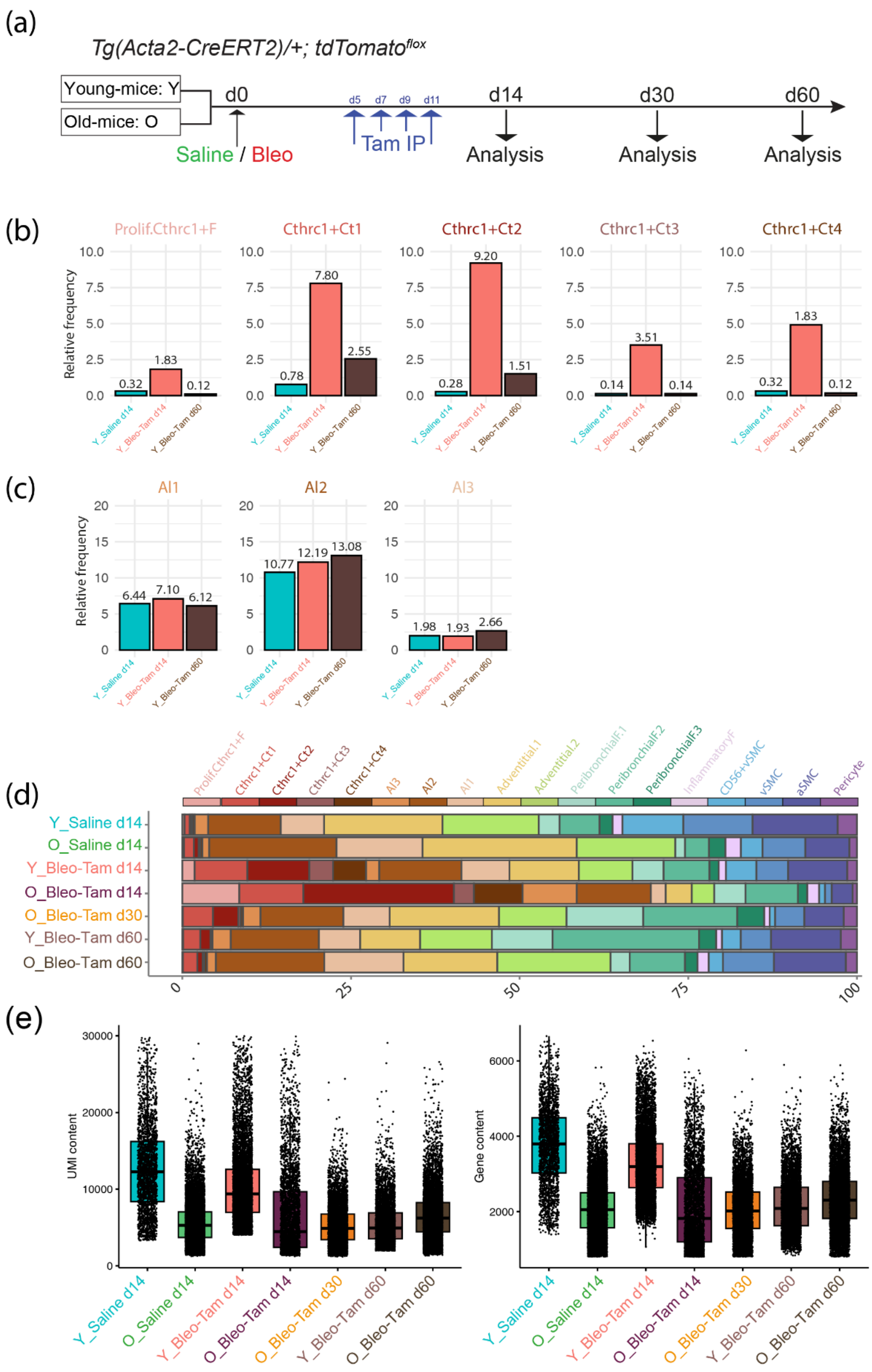
(a)
Tg(Acta2-CreERT2)/+; tdTomatoflox
Young-mice: Y
Old-mice: O
d0
d5 d7 d9 d11
d14
d30
d60
Saline / Bleo
Tam IP
Analysis
Analysis
Analysis
(b)
Prolif.Cthrc1+F
Cthrc1+Ct1
Cthrc1+Ct2
Cthrc1+Ct3
Cthrc1+Ct4
Relative frequency
0.32
1.83
0.12
0.78
7.80
2.55
0.28
9.20
1.51
0.14
3.51
0.14
0.32
1.83
0.12
(c)
AI1
AI2
AI3
Relative frequency
6.44
7.10
6.12
10.77
12.19
13.08
1.98
1.93
2.66
(d)
Y_Saline d14
O_Saline d14
Y_Bleo-Tam d14
O_Bleo-Tam d14
O_Bleo-Tam d30
Y_Bleo-Tam d60
O_Bleo-Tam d60
(e)
UMI content
Gene content

Figure S3**: Comparison of Cthrc1 and alveolar fibroblast subclusters in young versus old mice. (a)** Female *Tg(Acta2-CreERT2)/+;tdTomato*^{flox} young (8-12-week-old) and old (52-56-week-old) mice are either subjected to saline or 2 $U.kg^{-1}$ bleomycin (bleo). Saline lungs are collected at d14 and experimental lungs (Bleo-Tam) are collected at d14, d30, and d60 following bleomycin injury. **(b)** Relative frequencies of each *Cthrc1*-expressing subpopulation at each time-point in young mice. **(c)** Relative frequencies of each alveolar fibroblast subpopulation at each time point in young mice. **(d)** The relative frequencies of each subpopulation in each sample from the integrated dataset of young and old mice. **(e)** UMI and gene content in each sample from the integrated young and old mice dataset. O, old mice; Y, young mice

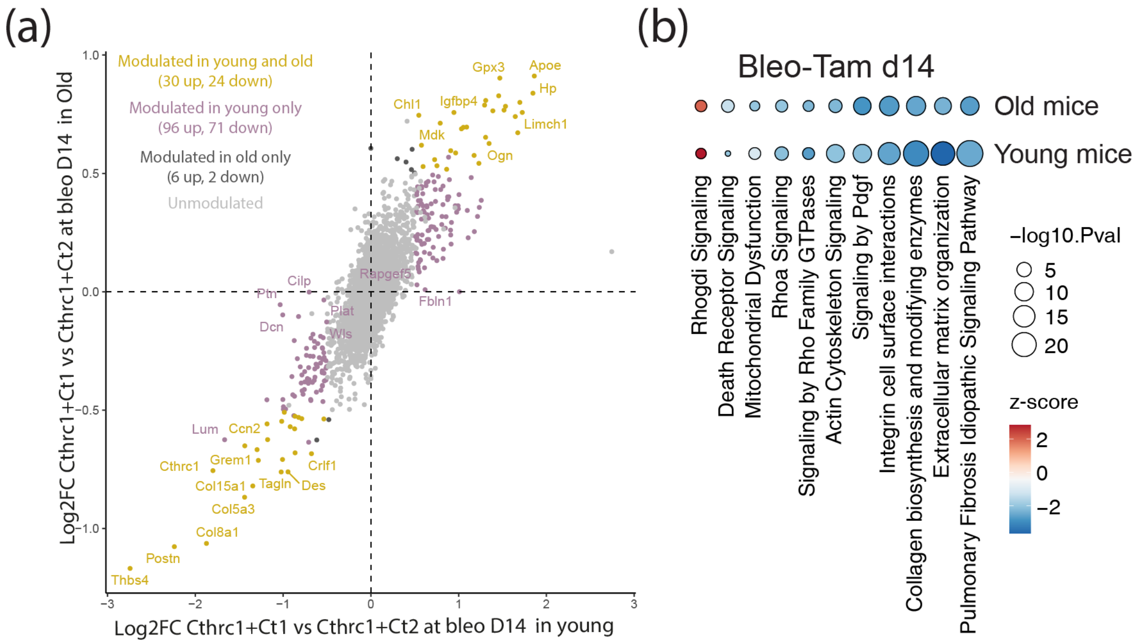

Figure S4: **Comparison of *Cthrc1*+ myofibroblast cells (Ct1 versus Ct2) during fibrosis formation (d14) in young and old mice. (a)** Correlation of the two Log2 Fold change obtained by comparing *Cthrc1*+ Ct1 to Ct2 of the Bleo-Tam d14 sample from young (8-12-week-old) and old (52-56-week-old) mice. Golden dots correspond to genes significantly modulated ($|log2FC| > 0.5$ and $p_{adj} < 0.05$) in both young and old. Mauve and dark gray dots correspond to genes only significantly modulated in young or old mice, respectively. **(b)** IPA canonical pathways enrichment obtained from the differential analyses of Ct1 compared to Ct2 cells of the Bleo-Tam d14 sample in both young and old mice.

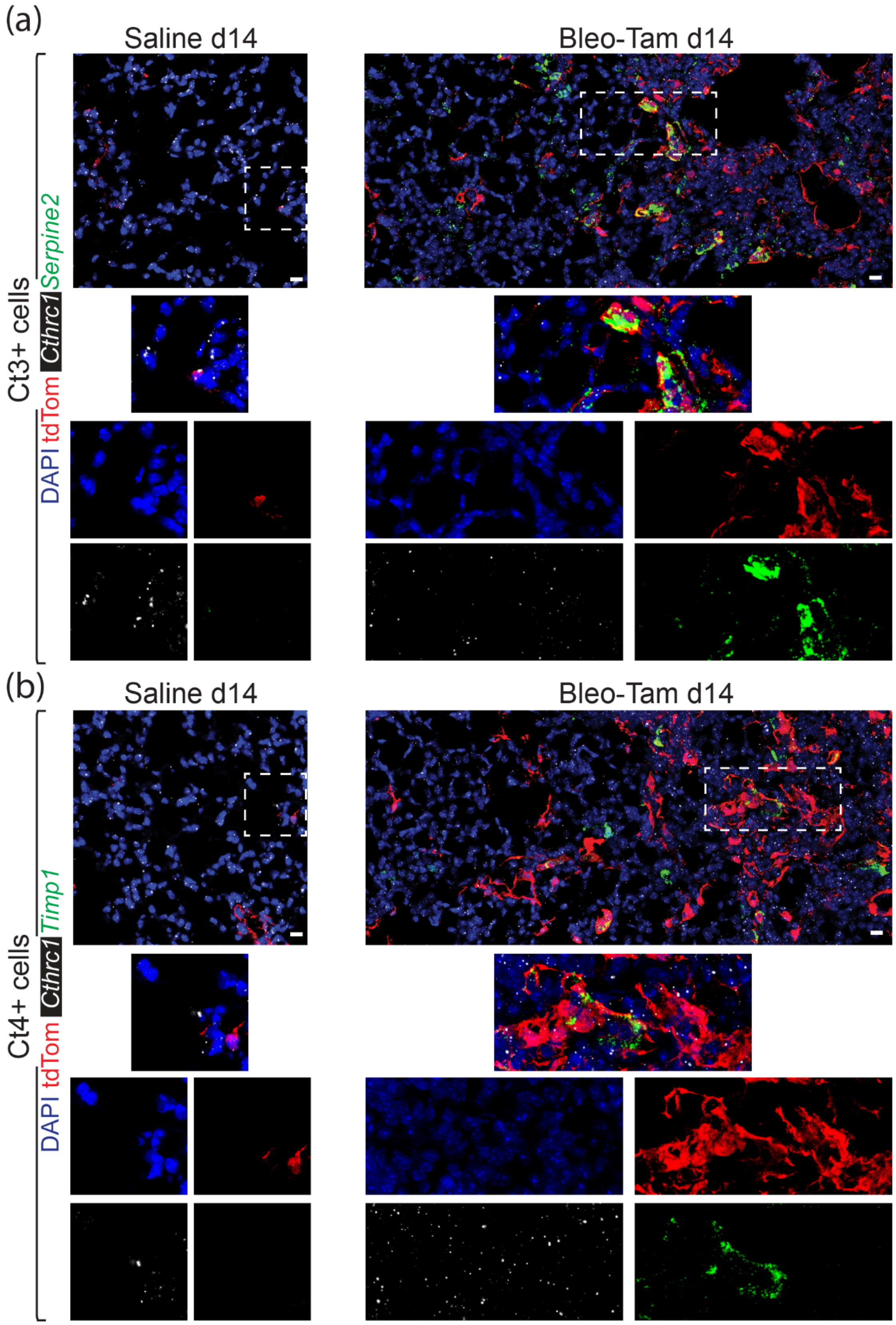
(a)
Saline d14
Bleo-Tam d14
Ct3+ cells
DAPI tdTom Cthrc1 Serpine2
(b)
Saline d14
Bleo-Tam d14
Ct4+ cells
DAPI tdTom Cthrc1 Timp1

Figure S5**: Characterization of Ct3 and Ct4 cells in saline and Bleo-Tam d14 lungs.** **(a)** RNA-scope of *Cthrc1*+aMYF marker *Cthrc1* and Ct3-cluster marker *Serpine2* showing colocalization with tdTom+ cells. **(b)** RNA-scope of *Cthrc1*+aMYF marker *Cthrc1* and Ct4-cluster marker *Timp1* showing colocalization with tdTom+ cells. Scale bars: (a, b): 150 µm.

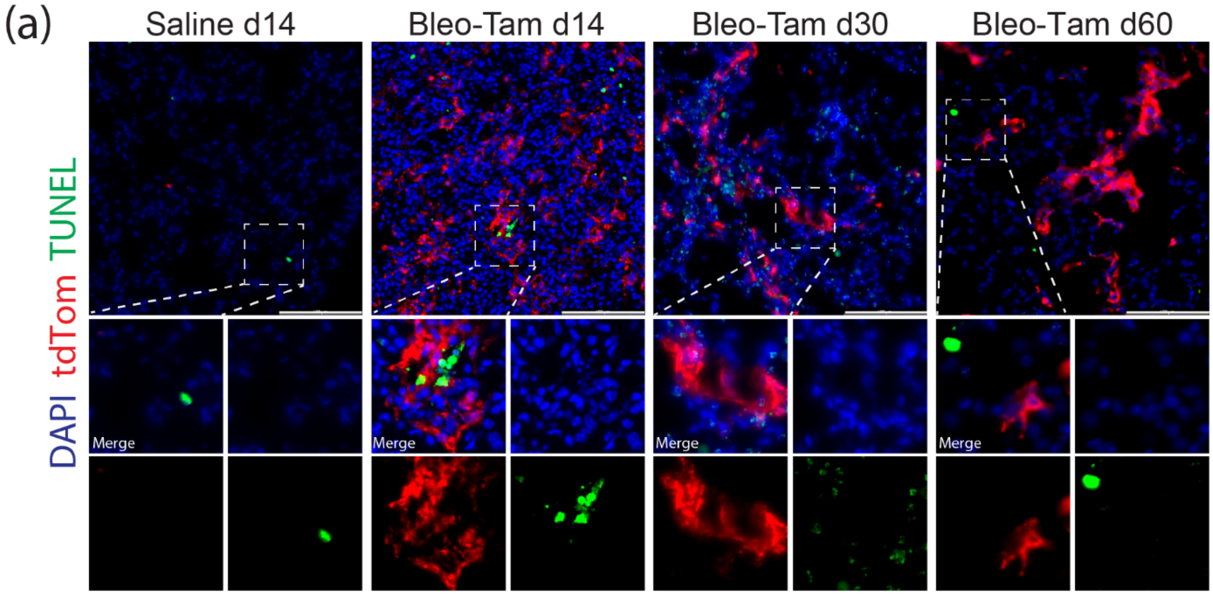

Figure S6**: TUNEL assay showing the tdTom+ cells undergo apoptosis during fibrosis formation (d14) and early fibrosis resolution (d30)**. Many tdTom+ cells persist during late fibrosis resolution (d60). Scale bar: 100 µm.
